# Multi-organ Dysfunction in Patients with COVID-19: A Systematic Review and Meta-analysis

**DOI:** 10.14336/AD.2020.0520

**Published:** 2020-05-13

**Authors:** Ting Wu, Zhihong Zuo, Shuntong Kang, Liping Jiang, Xuan Luo, Zanxian Xia, Jing Liu, Xiaojuan Xiao, Mao Ye, Meichun Deng

**Affiliations:** ^1^Department of Biochemistry and Molecular Biology & Hunan Province Key Laboratory of Basic and Applied Hematology, School of Life Sciences, Central South University, Hunan 410013, China.; ^2^Department of Cardiovascular Medicine, The Third Xiangya Hospital, Central South University, Changsha, Hunan 410013, China.; ^3^Xiangya School of Medicine, Central South University, Hunan 410013, China.; ^4^Hunan Yuanpin Cell Biotechnology Co., Ltd, Hunan 410129, China.; ^5^Department of Cell Biology, School of Life Sciences, Central South University, Changsha 410013, China.; ^6^Hunan Key Laboratory of Animal Models for Human Diseases, Hunan Key Laboratory of Medical Genetics & Center for Medical Genetics, School of Life Sciences, Central South University, Changsha 410013, China.; ^7^Molecular Science and Biomedicine Laboratory, State Key Laboratory for Chemo/Biosensing and Chemometrics, College of Biology, College of Chemistry and Chemical Engineering, Collaborative Innovation Center for Molecular Engineering for Theranostics, Hunan University, Changsha, China

**Keywords:** COVID-19, multiorgan dysfunction, acute multiorgan injury

## Abstract

This study aimed to provide systematic evidence for the association between multiorgan dysfunction and COVID-19 development. Several online databases were searched for articles published until May 13, 2020. Two investigators independently selected trials, extracted data, and evaluated the quality of individual trials. Single-arm meta-analysis was performed to summarize the clinical features of confirmed COVID-19 patients. Fixed effects meta-analysis was performed for clinically relevant parameters that were closely related to the patients’ various organ functions. A total of 73 studies, including 171,108 patients, were included in this analysis. The overall incidence of severe COVID-19 and mortality were 24% (95% confidence interval [CI], 20%-28%) and 2% (95% CI, 1%-3%), respectively. Patients with hypertension (odds ratio [OR] = 2.40; 95% CI, 2.08-2.78), cardiovascular disease (CVD) (OR = 3.54; 95% CI, 2.68-4.68), chronic obstructive pulmonary disease (COPD) (OR=3.70; 95% CI, 2.93-4.68), chronic liver disease (CLD) (OR=1.48; 95% CI, 1.09-2.01), chronic kidney disease (CKD) (OR = 1.84; 95% CI, 1.47-2.30), chronic cerebrovascular diseases (OR = 2.53; 95% CI, 1.84-3.49) and chronic gastrointestinal (GI) disease (OR = 2.13; 95% CI, 1.12-4.05) were more likely to develop severe COVID-19. Increased levels of lactate dehydrogenase (LDH), creatine kinase (CK), high-sensitivity cardiac troponin I (hs-cTnI), myoglobin, creatinine, urea, alanine aminotransferase (ALT), aspartate aminotransferase (AST), and total bilirubin were highly associated with severe COVID-19. The incidence of acute organ injuries, including acute cardiac injury (ACI); (OR = 11.87; 95% CI, 7.64-18.46), acute kidney injury (AKI); (OR=10.25; 95% CI, 7.60-13.84), acute respiratory distress syndrome (ARDS); (OR=27.66; 95% CI, 18.58-41.18), and acute cerebrovascular diseases (OR=9.22; 95% CI, 1.61-52.72) was more common in patients with severe COVID-19 than in patients with non-severe COVID-19. Patients with a history of organ dysfunction are more susceptible to severe conditions. COVID-19 can aggravate an acute multiorgan injury.

Coronaviruses are positive-stranded ribonucleic acid (+RNA) viruses belonging to the family *Coronaviridae* and order *Nidovirales* [1, 2]. Prior to December 2019, six coronavirus species had been identified and these primarily caused mild illness [2, 3]. However, in the past decades, two zoonotic coronaviruses, severe acute respiratory syndrome coronavirus (SARS-CoV) and Middle East respiratory syndrome coronavirus (MERS-CoV), resulted in severe and even fatal lower respiratory tract infections, with more than 8000 and 1500 confirmed cases and 10% and 37% case fatality rates (CFR), respectively [4, 5]. Although coronavirus infections have a huge effect, strategies to prevent and treat coronavirus infection are limited due to the lack of effective antiviral treatments [6].

At the beginning of December 2019, pneumonia due to an unknown cause was reported in a series of patients. On January 7, 2020, the cause was identified as a novel coronavirus infection. This virus was named SARS-CoV-2 by the World Health Organization (WHO). Similar to SARS-CoV and MERS-CoV, SARS-CoV-2 targets the respiratory tract and the dominant symptoms of COVID-19 at the beginning of the illness are fever, cough, fatigue, or myalgia [7, 8]. Although COVID-19 has a relatively low CFR, it is spreading rampantly, with more than four million confirmed cases, and it has affected the global economy and human health.

Studies have suggested that COVID-19 leads to the development of severe pneumonia, other complications, and even death, especially in high-risk patients [9, 10]. To date, neither a vaccine nor a specific treatment with a confirmed result has been available to patients. Hence, effective methods to improve the outcomes in patients with severe COVID-19 may include the early prevention of SARS-CoV-2 infection in high-risk patients and the early monitoring and intervention regarding the parameters associated with disease severity. It has been reported that patients with diabetes, hypertension, and coronary heart diseases are 2.85, 3.05, and 21.40 times more likely to have a poor prognosis, respectively, than that in patients without these diseases [10]. In addition, elevated alanine aminotransferase (ALT), lactate dehydrogenase (LDH), high-sensitivity cardiac troponin I (hs-cTnI), and urea levels have been reported to be associated with disease severity [11, 12]. The incidence of complications, including acute respiratory distress syndrome (ARDS) and acute cardiac injury (ACI), is higher in patients with severe COVID-19 [8, 10]. These clinically relevant parameters are closely related to various organ functions in patients. Hence, we wanted to know whether patients with a history of organ dysfunction are more susceptible to COVID-19 infection and whether COVID-19 infection aggravates acute damage to various organs. Given the rapid spread of COVID-19 with no specific treatment available, it is urgent to analyze published and high-quality clinical studies to identify guidelines for the management of the patients. This study aimed to provide systematic evidence for the association between multiorgan dysfunction and COVID-19 severity and to compare differences in the indices of organ function among COVID-19, SARS, and MERS. In addition, based on the existing literature, we have provided certain treatment suggestions for patients with dysfunction of various organs.

## MATERIALS AND METHODS

This study was registered in PROSPERO, with registration No. CRD42020177984.

### Search strategies

Studies published in the EMBASE, PubMed, Web of Science, MedRxiv, and Biorxiv databases before or on May 13, 2020, were searched using the following search terms: “SARS-2-CoV,” “coronavirus,” “COVID19,” “2019-nCoV,” “clinical features,” “clinical characteristics,” “clinical outcomes,” “cardiac diseases,” “renal diseases,” “pulmonary diseases,” “liver diseases,” “neurological disease,” “gastrointestinal (GI) disease,” “nervous system,” and “digestive system,” alone or in combination, without language restriction.

### Eligibility criteria

Eligible studies were included in the meta-analysis according to the following criteria: (1) type of participants: patients (≥18 years old) in each study who were diagnosed as having COVID-19 and (2) type of study: all studies that provide information about symptoms, medical history, laboratory results, and outcomes of COVID-19 patients. Studies that enrolled severe disease and non-severe disease groups (or ICU and non-ICU or death and survival) were included to examine the relationship between organ dysfunction and COVID-19 severity. Trials were excluded if any of the following factors were identified: (1) study design: comments, letters, case reports, abstracts, and reviews; (2) type of participants: animals, patients <18 years old, and pregnant women; and (3) insufficient information concerning evaluation rates.

### Trial selection

After eliminating duplicates, two independent investigators reviewed the remaining identified trials to confirm that they fulfilled the inclusion criteria. Finally, the reference lists of the included studies were screened to examine other potentially relevant studies. All the disagreements were discussed and solved after rechecking the source data with the third investigator; in all the cases, one person recognized an error.

### Data extraction and quality assessment

Two reviewers extracted data independently using a predefined data extraction form. The data extracted included the last name of the first author, publication year, sample size (n), mean age (years), percentage of female patients (%), study design, geographical region, overall cases (n), symptoms, comorbidities, laboratory findings, and complications. The quality assessment forms recommended by the Agency for Healthcare Research and Quality were used for quality assessment of the included trials. We resolved all disagreements through a discussion.

### Statistical analyses

The weighted mean difference (WMD) and odds ratio (OR) were used to compare continuous and dichotomous variables, respectively. All the results were reported with 95% confidence intervals (CIs). Median (range) or median (interquartile range [IQR]) will be converted to the form of mean (standard deviation [SD]) [13]. We pooled the effect estimates of the outcomes by using fixed-effect models. A random effect model was used when significant heterogeneity was detected. Heterogeneity was assessed by using the *I*^2^ value, and an *I*^2^ of >50% was considered significant. The sensitivity analyses were made by excluding one study at a time to observe the change of the effects on the outcomes. Egger’s test, and Begg’s test (P < 0.10) were used to suggest the possible publication bias of outcomes. We performed all statistical analyses with the STATA 12.0 statistical software package (StataCoporation, College Station, Texas, USA).

**Table 1:** Clinical features of COVID-19 cases.

	Overall		Overall
**CFR, % (95%CI)**	2(1-3)	**Laboratory results (95%CI)**	
**CSR, % (95%CI)**	24(20-28)	**Liver function**	
**Comorbidity, % (95%CI)**		ALT, U/L	27.02(24.67-29.37)
Hypertension	19(15-23)	AST, U/L	30.50(28.09-32.91)
Diabetes	10(8-13)	Total bilirubin, mmol/L	11.10(10.16-12.04)
CVD	8(4-14)	**Renal function**	
COPD	2(2-3)	Creatinine, μmol/L	69.18(65.22-73.13)
Cerebrovascular diseases	3(2-5)	Urea mmol/L	4.76(4.31-5.22)
CLD	3(2-4)	**Cardiac function**	
CKD	3(1-4)	Myoglobin, ng/mL	49.96(37.11-62.81)
Tumor	3(1-5)	CK, U/L	84.69(74.51-94.86)
GI disease	7(5-8)	LDH, U/L	275.43(247.82-303.04)
Nervous system disease	1(0-2)	hs-cTnI, pg/mL	13.08(8.22-17.94)
**Neurologic symptoms, % (95%CI)**		**CT % (95%CI)**	
Headache	15(9-23)	Bilateral involvement of chest radiographs	85(78-90)
Dizziness	10(7-13)	**Complications% (95%CI)**	
Impaired consciousness	9(8-10)	ARDS	12(7-18)
Olfactory and/or taste disorders.	54(37-70)	ACI	6(3-9)
Olfactory disorders	28(9-52)	Shock	6(2-11)
Taste disorders	30(12-52)	AKI	4(1-7)
**GI symptoms, % (95%CI)**		Acute cerebrovascular disease	2(1-3)
Anorexia	21(14-29)	**GI symptoms, % (95%CI)**	
Abdominal pain	5(2-10)	Nausea	7(4-11)
Diarrhea	9(6-12)	Vomiting	5(1-10)

Abbreviations: ACI: acute cardiac injury; AKI: acute kidney injury; ALT, alanine aminotransferase; ARDS, acute respiratory distress syndrome; AST, aspartate aminotransferase; CFR, case fatality rate; CI: confidence interval; CK: creatine kinase; CKD: chronic kidney diseases; CLD: chronic liver diseases; COPD, chronic obstructive pulmonary disease; CSR, case severity rate; CVD: cardiovascular diseases; GI: gastrointestinal; hs-cTnI: high-sensitivity cardiac troponin I; LDH: lactate dehydrogenase;

## RESULTS

### Selection of included studies and Study Characteristics

**Figure 1.**
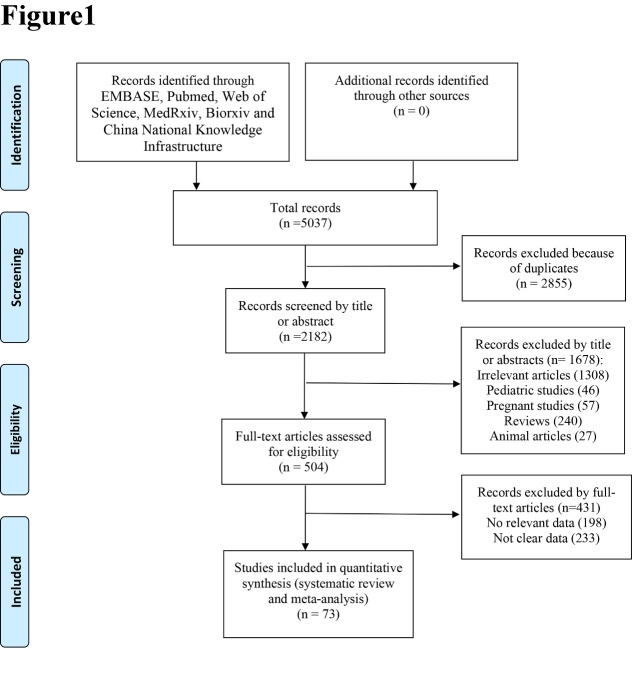
PRISMA Diagram of Study Selection.

A total of 5037 relevant articles were identified by searching several online databases. Figure 1 presents the screening and selection process of the eligible trials. The characteristics of the included trials are listed in Supplementary Table 1. This meta-analysis included 73 studies [8, 11, 12, 14-83]. Among these studies, 54 were from China, 4 from Italy, 3 from The United States, 2 from The United Kingdom, 2 from France, 2 from Spain, and the remaining 6 from other countries. Clinical features of severe and non-severe COVID-19 cases were reported by 48 studies. The results of the quality assessments were presented in Supplementary Table 1.

### Clinical features in patients with COVID-19

The results of this meta-analysis revealed that the overall CFR was 2% (95% confidence interval [CI], 1%-3%) and the proportion of patients with severe illness was 24% (95% CI, 20%-28%). The most common comorbidities were hypertension 19% (95% CI, 15%-23%), followed by diabetes 10% (95% CI, 8%-13%) and cardiovascular disease (CVD) 8% (95% CI, 4%-14%). The prevalence of chronic obstructive pulmonary disease (COPD), cerebrovascular disease, chronic liver disease (CLD), chronic kidney disease (CKD), tumor, chronic GI disease and nervous system disease were 2% (95% CI, 2%-3%), 3% (95% CI, 2%-5%), 3% (95% CI, 2%-4%), 3% (95% CI, 1%-4%), 3% (95% CI, 1%-5%), 7% (95% CI, 5%-8%), and 1% (95% CI, 0%-2%), respectively. Furthermore, we analyzed the prevalence of complications caused by COVID-19. The most common complications were ARDS 12% (95% CI, 7%-18%), followed by ACI 6% (95% CI, 3%-9%) and shock 6% (95% CI, 2%-11%). The overall proportion of acute kidney disease (AKI) and acute cerebrovascular disease was 4% (95% CI, 1%-7%) and 2% (95% CI, 1%-3%), respectively. The laboratory results revealed that cardiac function indexes, such as LDH (275.43 U/L; 95% CI, 247.82-303.04 U/L) and myoglobin (49.96 ng/mL; 95% CI, 37.11-62.81. ng/mL) levels were elevated. The pooled results of the included studies showed that the most common neurologic symptoms were olfactory and/or taste disorders (54%; 95% CI, 37%-70%), and the most dominant GI symptom was anorexia (21%; 95% CI, 14%-29%) (Table 1). Notably, olfactory and/or taste disorders were less common in Asia. The pooled results were from the included studies which were outside of Asia.

**Figure 2.**
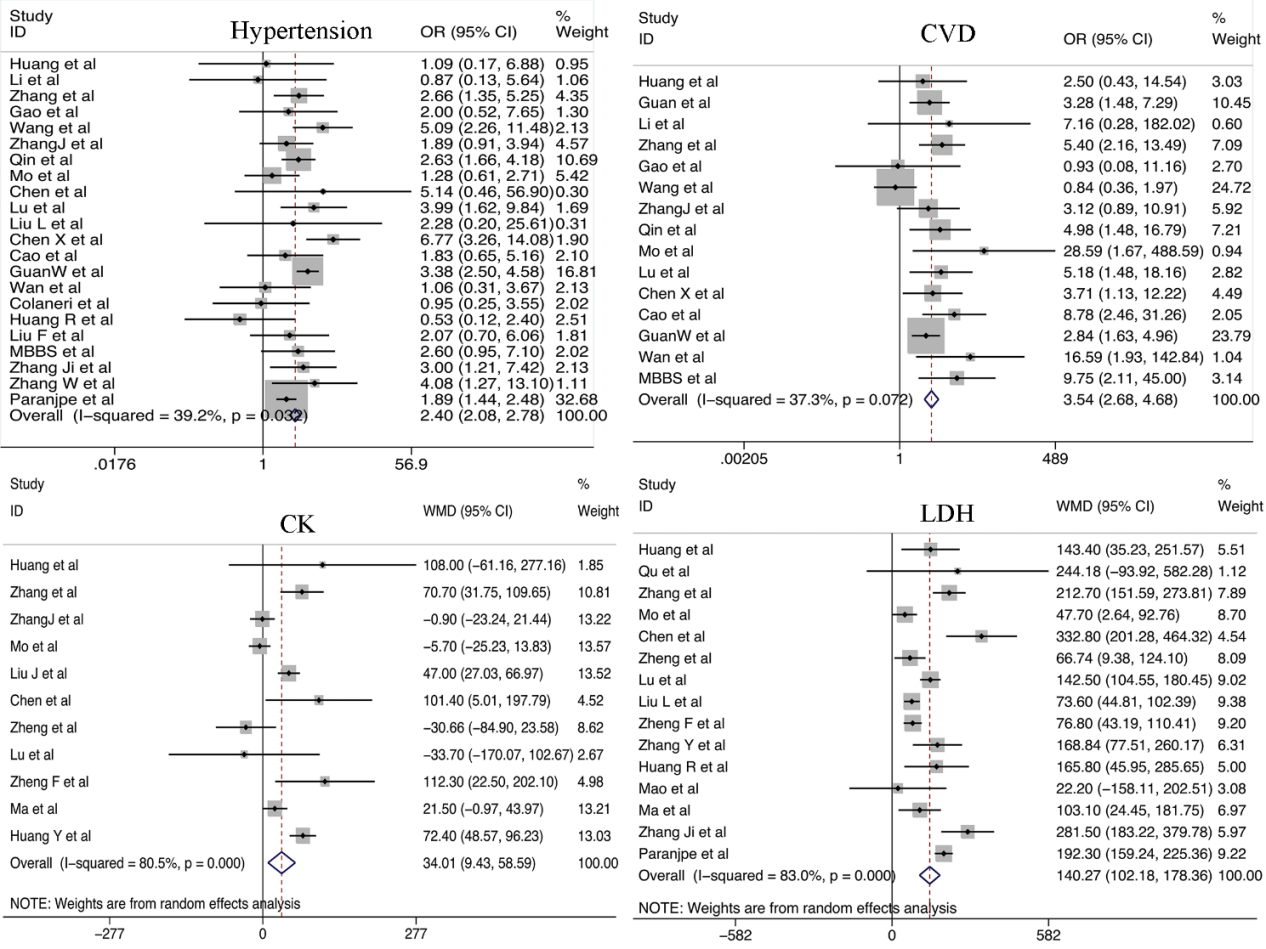
Relationship between cardiac dysfunction (Hypertension, CVD, CK and LDH) and COVID-19 severity.

### Organ dysfunction and COVID-19 severity

#### Cardiac dysfunction

**Figure 3.**
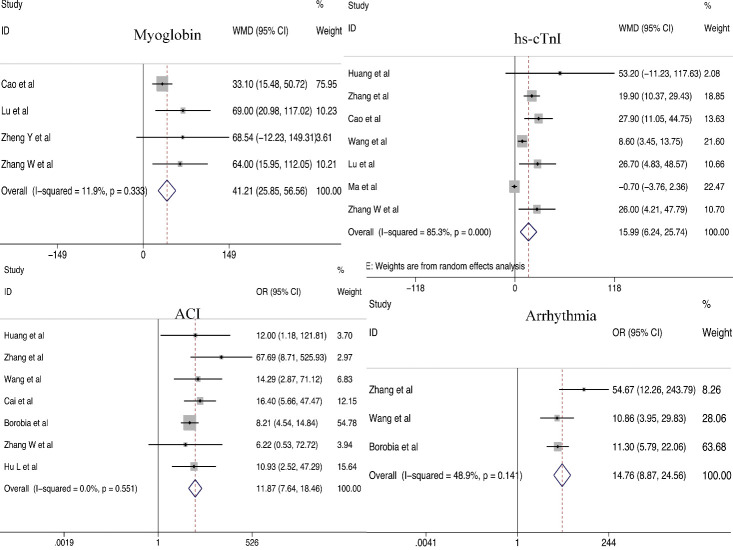
Relationship between cardiac dysfunction (hs-cTnI, myoglobin, ACI and arrhythmia) and COVID-19 severity.

We analyzed the relationship between cardiac dysfunction and the development of COVID-19. Disease severity was found to be closely associated with the history of hypertension (odds ratio [OR] = 2.40; 95% CI, 2.08-2.78) and CVD (OR = 3.54; 95% CI, 2.68-4.68). Obvious differences in LDH (weighted mean difference [WMD] = 140.27 U/L; 95% CI, 102.18-178.36 U/L), CK (WMD =34.01 U/L; 95% CI, 9.46-58.59 U/L), hs-cTnI (WMD =15.99 pg/mL; 95% CI, 6.24-25.74 pg/mL), and myoglobin (WMD = 41.21 ng/mL; 95% CI, 25.85-56.56. ng/mL) levels were noted between patients with severe and non-severe COVID-19. In addition, a significantly higher incidence of ACI was observed among patients with severe COVID-19 than that among patients with non-severe COVID-19 (OR = 11.87; 95% CI, 7.64-18.46). Patients with severe COVID-19 were more likely to experience arrhythmias (OR = 14.76; 95% CI, 8.87-24.56). In addition, we found preexisting diabetes was positively associated with COVID-19 severity (OR = 2.25; 95% CI, 1.91-2.65) (Fig. 2 and 3 and supplemtary Fig. 1).

#### Treatment suggestions

In the preclinical *in vitro* studies, chloroquine (CQ) was found to inhibit SARS-CoV-2 activity [84]. Hydroxychloroquine (HCQ), a derivative of CQ, was found to be more potent than CQ *in vitro*. In addition, researchers have confirmed the clinical benefits of CQ for SARS [85]. A randomized clinical trial found that HCQ significantly shortened the time to clinical recovery and promoted pneumonia absorption among COVID-19 patients [86]. Therefore, the guidelines recommend that the administration of CQ/HCQ may help to improve the clinical outcomes of COVID-19 patients. However, the efficiency and safety of CQ and HCQ for treating COVID-19 remain uncertain due to recent controversial results [87], especially its’ potential cardiac toxicity. CQ/HCQ alone can cause QT prolongation and is known to cause torsades de pointes, especially among patients with a history of cardiac dysfunction and when administered in combination with other drugs such as lopinavir/ritonavir (LPV/r); [88]. It was reported that 30% of patients treated with HCQ + azithromycin developed new QT prolongation of > 40 ms [89]. Therefore, we strongly recommend close monitoring of electrocardiograms with QTc evaluation and correction of any electrolyte imbalance prior to administering CQ/HCQ [90]. Non-essential QT-prolonging drugs, including LPV/r, should be avoided in patients with a history of cardiac dysfunction.

#### Renal dysfunction

**Figure 4.**
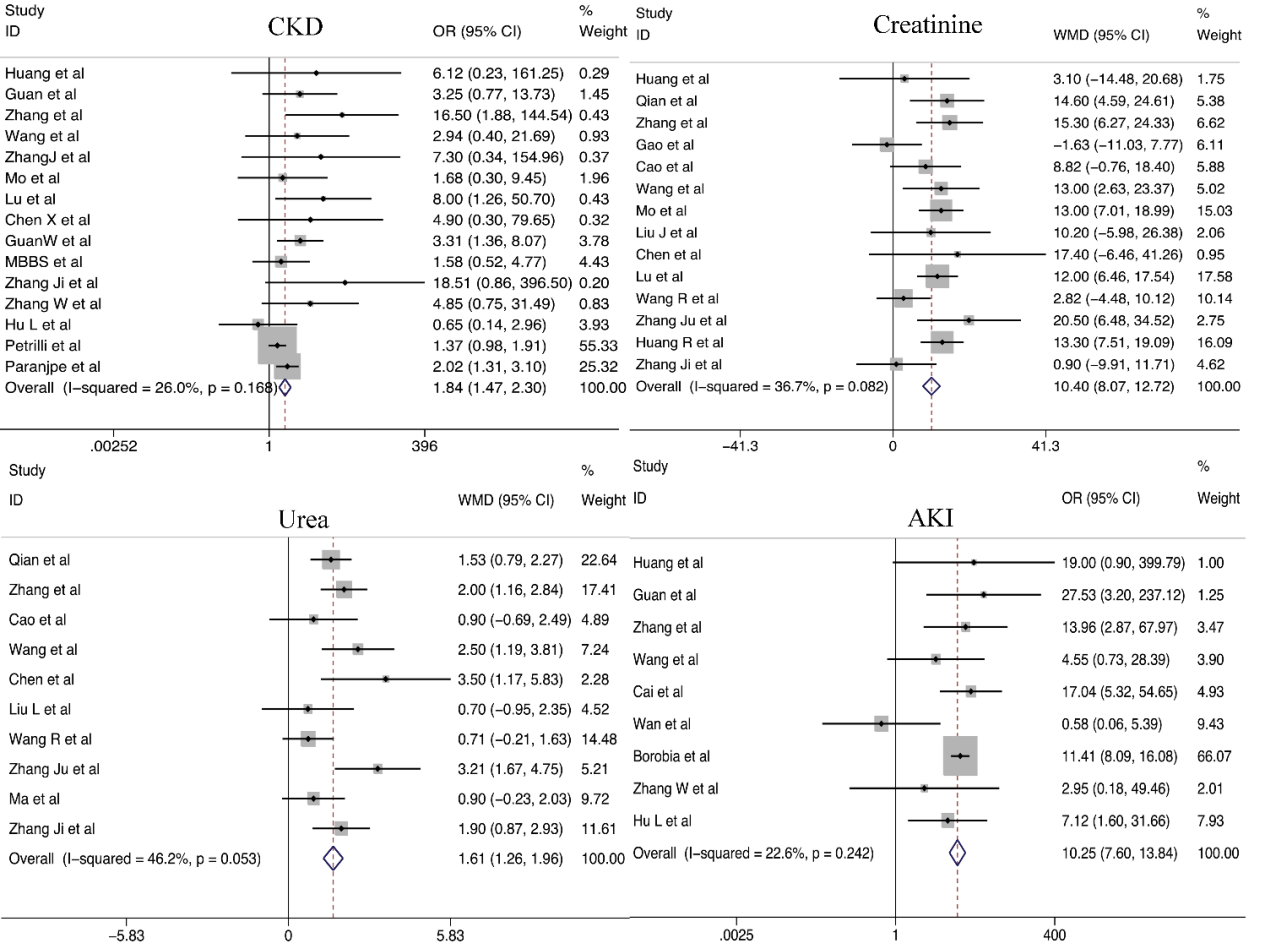
Relationship between renal dysfunction and COVID-19 severity.

The pooled results indicated that patients with CKD were at a high risk of developing severe COVID-19 (OR = 1.84; 95% CI, 1.47-2.30). Patients with severe COVID-19 had higher levels of creatinine (WMD = 10.40 μmol/L; 95% CI, 8.07-12.72 μmol/L) and urea (WMD = 1.61 mmol/L; 95% CI, 1.26-1.96mmol/L) than those in patients with non-severe COVID-19. Furthermore, because severe infection aggravates kidney damage, AKI was more likely to occur among patients with severe COVID-19 than among patients with non-severe COVID-19 (OR=10.25; 95% CI, 7.60-13.84) (Fig. 4).

#### Treatment suggestions

The present study revealed that the overall proportion of AKI was 4% (95% CI, 1%-7%) and AKI was associated with in-hospital mortality of COVID-19 patients [91]. According to the latest treatment protocol (Pilot Version 7) issued by the Chinese Health Commission, continuous renal replacement therapy (CRRT) is recommended for patients with severe COVID-19 and renal damage, especially for those with stage 3 AKI. CRRT removes inflammatory cytokines to regulate renal metabolic adaptation and improves kidney energy balance to protect the kidneys in patients with AKI [92]. Wang et al. suggested that COVID-19 patients with renal dysfunction should be monitored regularly. Once acute kidney failure develops, potential interventions, including CRRT, should be immediately initiated to protect renal function [93]. However, CRRT intensity should be carefully determined. A study indicated that higher-intensity CRRT treatment did not reduce mortality in patients with severe COVID-19 and AKI as compared with that in the control group [94].

#### Pulmonary dysfunction

**Figure 5.**
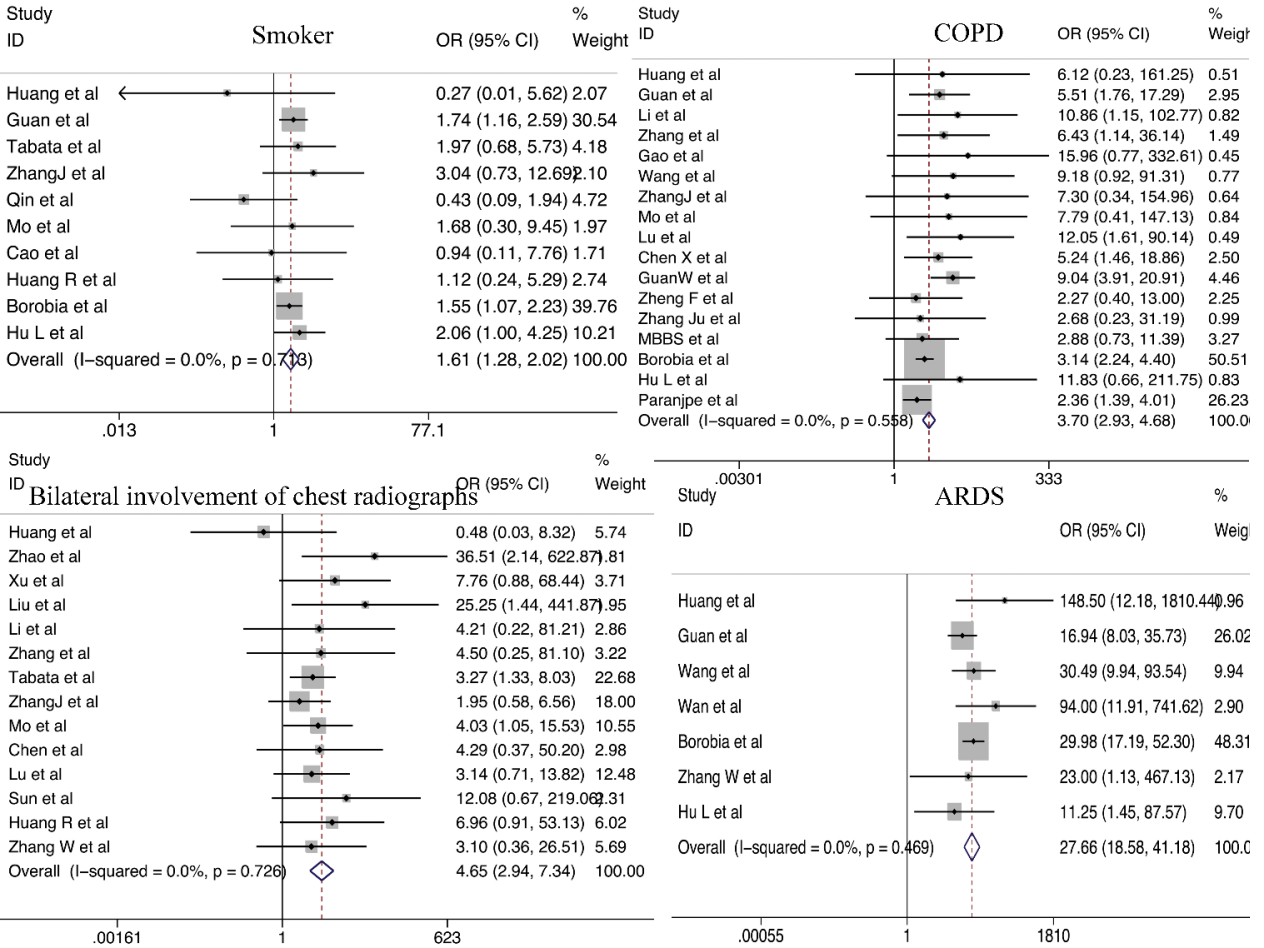
Relationship between pulmonary dysfunction and COVID-19 severity

COPD (OR = 3.70; 95% CI, 2.93-4.68) and smoking (OR = 1.61; 95% CI, 1.28-2.02) were significant predictors of severe COVID-19. Patients who presented with bilateral involvement on chest radiography on admission were four times more likely to develop severe COVID-19 (OR = 4.65; 95% CI, 2.94-7.34). Finally, patients with severe COVID-19 were found to have a significantly higher risk of developing ARDS compared to that in those with non-severe COVID-19 (OR=27.66; 95% CI, 18.58-41.18). (Fig. 5).

#### Treatment suggestion

Corticosteroid treatment is a double-edged sword and its’ effectiveness in treating COVID-19 remains controversial. One study indicated that delayed viral clearance was not observed in patients treated with low dose corticosteroids, while another trial revealed that patients treated with a corticosteroid demonstrated more abnormalities on chest CT and more clinical symptoms [95, 96]. ARDS, the leading cause of death in patients with SARS and MERS, is also a challenge in COVID-19 patients. ARDS is closely associated with overwhelming inflammation and cytokine-related lung injury. A retrospective cohort study revealed that methylprednisolone treatment appears to reduce the risk of death in COVID-19 patients with ARDS (HR = 0.38; 95% CI, 0.20-0.72) [97]. Given the urgent clinical demand, corticosteroid treatment is weakly recommended for COVID-19 patients with ARDS due to the low-quality evidence. However, it is not recommended for routine treatment [98].

#### Liver dysfunction

**Figure 6.**
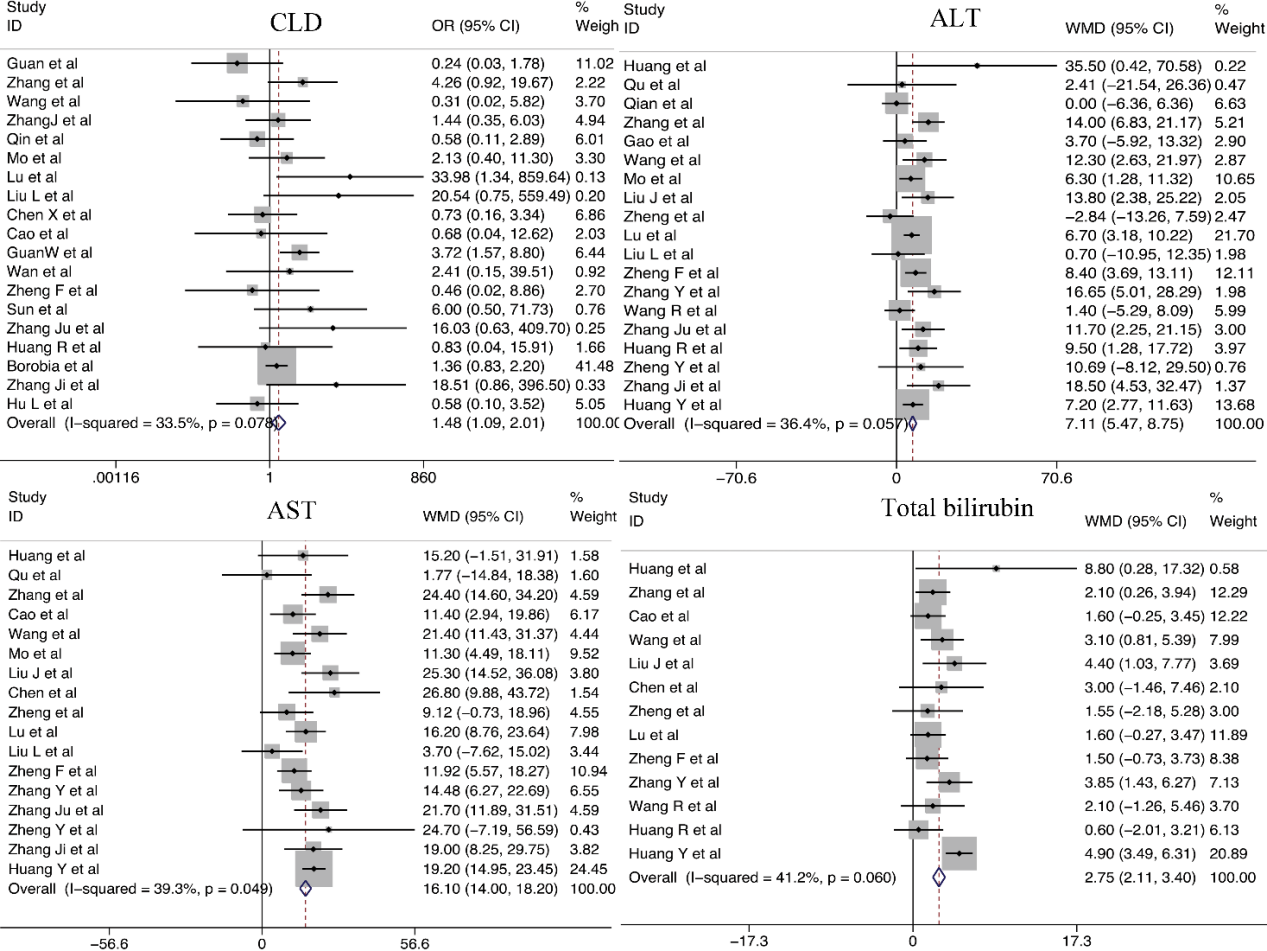
Relationship between liver dysfunction and COVID-19 severity

We analyzed the relationship between liver dysfunction and COVID-19 severity. CLD was a significant predictor of COVID-19 severity (OR = 1.48; 95% CI, 1.09-2.01). Significant differences in ALT (WMD = 7.11 U/L; 95% CI, 5.47-8.75 U/L), aspartate aminotransferase (AST; WMD = 16.10 U/L; 95% CI, 14.00-18.20 U/L), and total bilirubin (WMD = 2.75 mmol/L; 95% CI, 2.11-3.40 mmol/L) were observed in patients with severe and non-severe COVID-19. One study specifically presented a higher incidence of acute liver injury in patients with severe COVID-19 as compared with that in patients with non-severe COVID-19. This is consistent with laboratory indicators [29] (Fig. 6).

#### Treatment suggestion

One study found that SARS patients treated with LPV/r appeared to have a milder disease course, better outcomes, and significantly reduced viral loads [99]. Similarly, lopinavir has activity against MERS-CoV [100, 101]. As LPV/r is effective against other coronaviruses, researchers were optimistic to prescribe LPV/r for the treatment of COVID-19. However, a randomized, controlled, open-label trial conducted in China revealed that no significant difference was observed between LPV/r treatment and the control group of patients with severe COVID-19 in clinical improvement, reduced mortality, and/or changes in viral load [102]. The liver biopsy results of fatal COVID-19 patients indicated that the possibility of drug-induced acute liver injury could not be ruled out [103]. A study identified that patients with liver damage were administered a higher rate of LPV/r than that in those with normal liver function [104]. Hence, we do not recommend LPV/r as a routine treatment option for patients with a history of liver dysfunction. Further, clinicians should closely monitor liver-function indicators even in patients with normal liver function, if they are being treated with LPV/r.

#### Neurologic dysfunction

**Figure 7.**
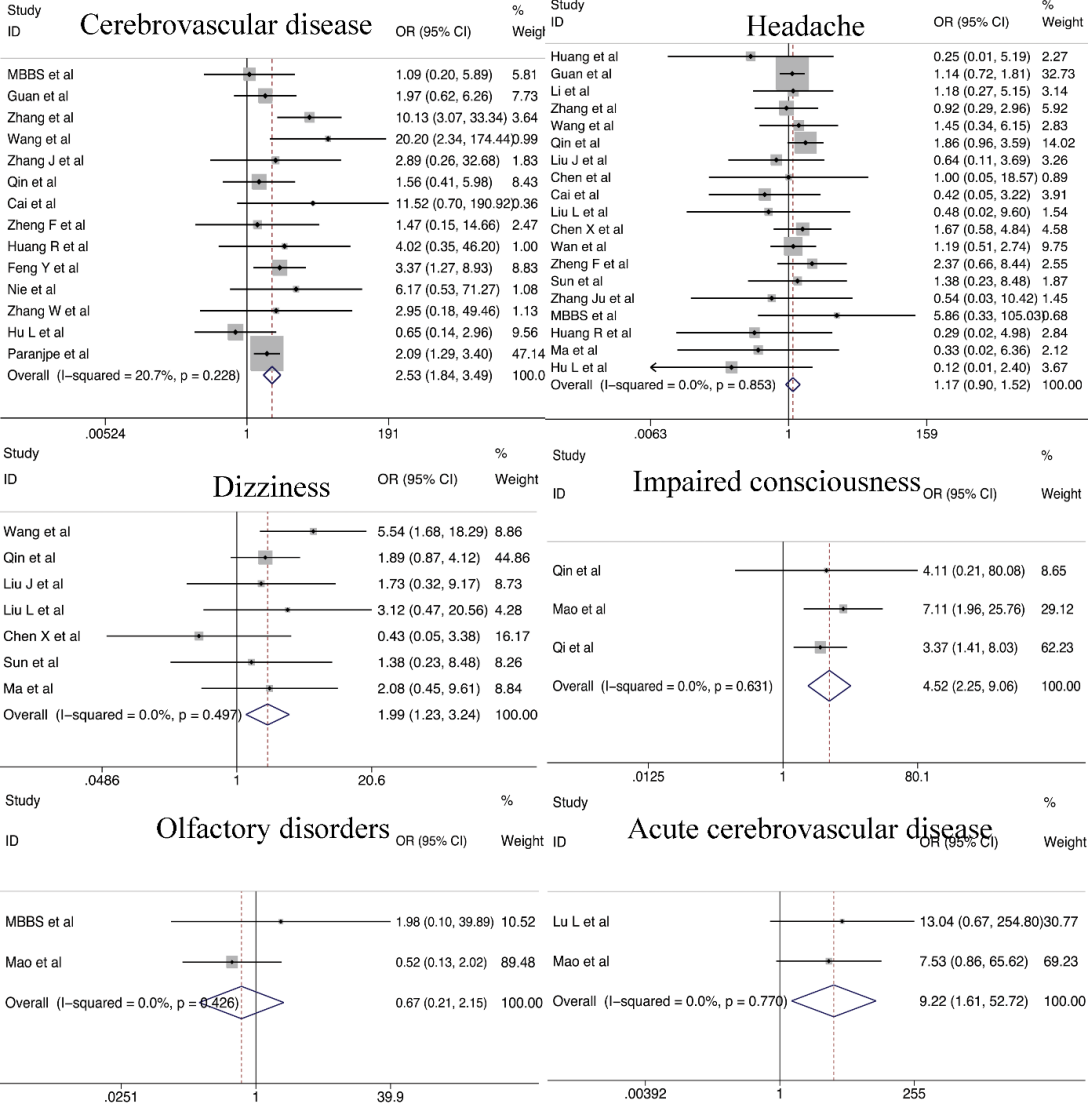
Relationship between neurologic dysfunction and COVID-19 severity.

Patients with chronic cerebrovascular diseases tended to face a higher risk of developing severe COVID-19 compared to those without chronic cerebrovascular diseases (OR = 2.53; 95% CI, 1.84-3.49). Patients with severe COVID-19 were more likely to have nervous system manifestations, such as dizziness (OR = 1.99; 95% CI, 1.23-3.24) and impaired consciousness (OR = 4.52; 95% CI, 2.25-9.06). Few differences in the incidence of headache (OR = 1.17; 95% CI, 0.90-1.52) and olfactory disorders (OR = 0.67; 95% CI, 0.21-2.15) were noted between patients with severe and non-severe COVID-19. Acute cerebrovascular diseases were significantly more common in severe infection compared with non-severe infection (OR = 9.22; 95% CI, 1.61-52.72) (Fig. 7)

#### Treatment suggestions

Ischemic stroke has been recognized as a complication in patients with COVID-19, especially in patients with severe infection [105]. However, the precise mechanisms are not yet clear. Obviously, an elevated D-dimer level was noted in patients with COVID-19 who developed stroke, indicating a hypercoagulable state [106]. It was suggested that production of antiphospholipid antibodies (aPL) accompanying COVID-19 infection may be closely associated with both venous and arterial thrombotic events [107]. The mortality of COVID-19 patients with stroke was much higher than that of patients with stroke alone [106, 108]. Considering the high mortality and prothrombotic state, it seems reasonable to initiate treatment with anticoagulants such as low molecular weight heparin (LMWH) and a recombinant tissue plasminogen activator (rt-PA) [109]. Compared with antiplatelet treatment, patients treated with LMWH had lower mortality [106]. Notably, patients who developed stroke were more likely to have multiple organ dysfunction [106]. Patients with hepatic dysfunction may have a significant reduction in their ability of hepatic clearance, which would increase the risk of intracranial hemorrhage (ICH). In addition, leukocytosis, elevated C reactive protein levels and D-dimer, which were commonly seen in COVID-19 patients, were identified as risk factors of ICH among patients without COVID-19 infection [110]. Therefore, it is reasonable to assess the coagulation profile for COVID-19 patients with acute ischemic stroke prior to administration of rt-PA and LMWH to determine the risk benefit ratio [109].

#### Gastrointestinal dysfunction

**Figure 8.**
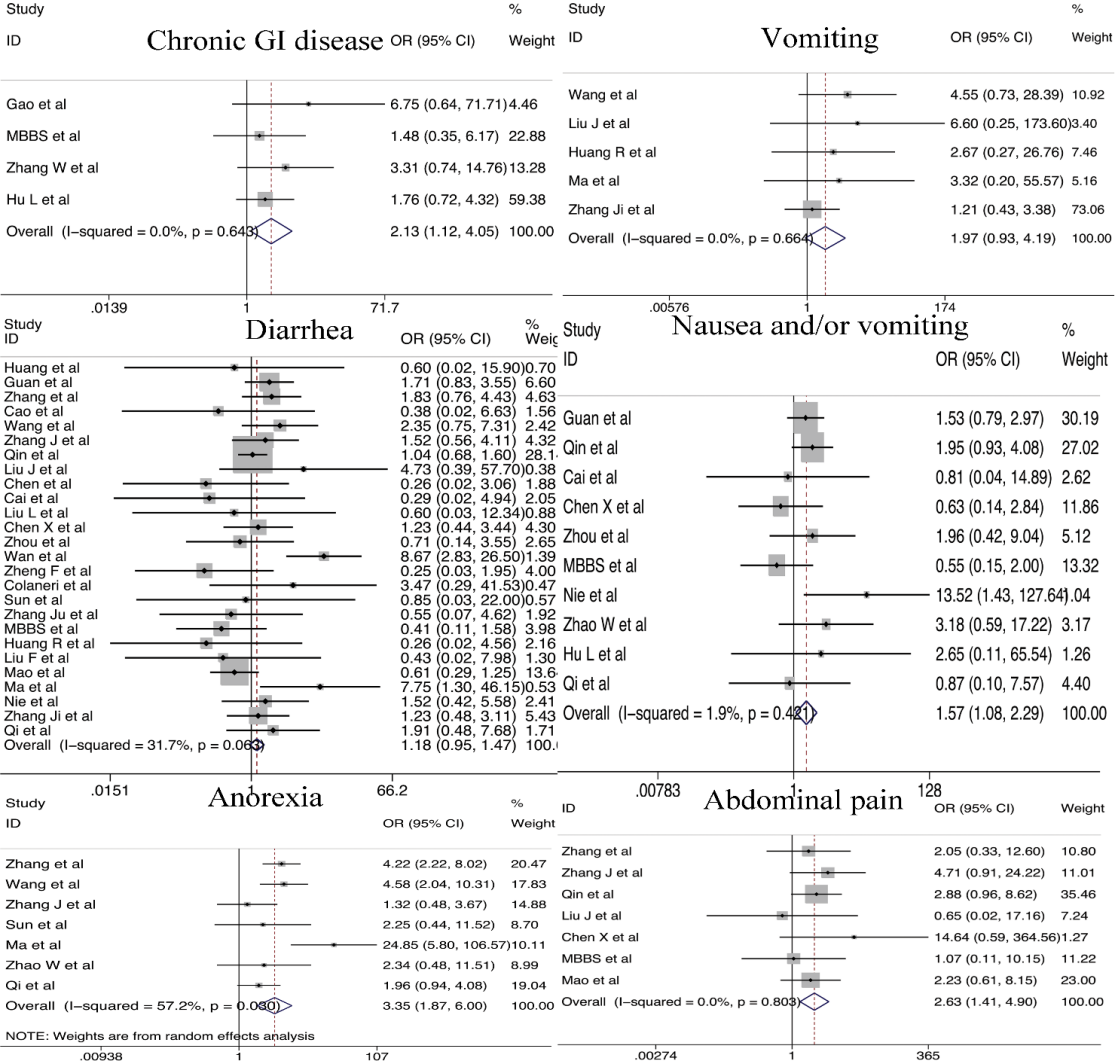
Relationship between gastrointestinal dysfunction and COVID-19 severity.

Chronic GI diseases were closely associated with severe infection (OR = 2.13; 95% CI, 1.12-4.05). GI symptoms, including nausea and/or vomiting (OR=1.57; 95% CI, 1.08-2.29), anorexia (OR = 3.35; 95% CI, 1.87-6.00) and abdominal pain (OR = 2.63; 95% CI, 1.41-4.90) were more likely to be observed in severe COVID-19 patients compared with non-severe COVID-19 patients. However, the incidence of diarrhea (OR=1.18; 95% CI, 0.95-1.47) and vomiting (OR=1.97; 95% CI, 0.93-4.19) were similar in patients with severe and non-severe COVID-19 (Fig. 8).

#### Treatment suggestion

As the COVID-19 pandemic expands, the impact of COVID-19 on patients with inflammatory bowel disease (IBD) has been a major concern. A significant number of IBD patients require immunosuppressors that target extensive inflammation. This may weaken the immune system and predispose IBD patients to a higher risk of COVID-19 infection. However, according to evidence emerging from China, Italy and other counties, there was no obvious indication that IBD patients were at greater risk of COVID-19 infection compared to the general population [111-113]. Thiopurines were related to a risk of serious viral infection in IBD patients, while a meta-analysis revealed that anti-tumor necrosis factor therapy was associated developing an opportunistic infection [114, 115]. However, discontinuation of IBD drugs should not be recommended for IBD patients during the COVID-19 pandemic [111]. This was supported by the results of the first case series report of COVID-19 infection in IBD patients [116]. Clinicians should be alert regarding patients treated with immunosuppressors and carefully monitor the presence of symptoms associated with COVID-19 [117]. The withholding of ongoing therapies should be considered only in confirmed COVID-19 patients. Therapies must be assessed carefully according to the balance between the severity of COVID-19 and the status of IBD [118].

**Table 2:** Comparison of impacts of organ function on severity of COVID-19, MERS and SARS.

	OR/WMD, 95%CI		
	COVID-19	SARS	MERS
**Cardiac function**			
Hypertension	OR = 2.40; 95% CI, 2.08-2.78		OR^138^ = 2.6; 95% CI, 2.2-3.5
CVD	OR = 3.54; 95% CI, 2.68-4.68	OR = 7.42; 95% CI, 4.34-12.69	OR^138^ = 3.5; 95% CI, 3.1-4.8
LDH U/L	WMD = 140.27; 95% CI, 102.18-178.36	WMD = 214.14; 95% CI, 31.09-217.19	
CK, U/L	WMD = 34.01; 95% CI, 9.46 -58.59	WMD = 149.91; 95% CI, 94.03-205.79	
**Renal function**			
CKD	OR = 1.84; 95% CI, 1.47-2.30	OR = 7.82; 95% CI, 3.52-17.38	OR^138^= 2.2; 95% CI, 1.7-4.9
Creatinine, μmol/L	WMD = 10.40; 95% CI, 8.07 - 12.72	WMD = 10.31; 95% CI, 6.76 - 13.85	WMD = 109.92; 95% CI, 21.33 to 198.5
Urea mmol/L	WMD = 1.61; 95% CI, 1.26 -1.96	WMD = 2.52; 95% CI, 1.26 - 3.78	WMD = 7.63; 95% CI, 6.52-8.74
**Pulmonary function**			
Smoker	OR=1.61; 95% CI, 1.28-2.02		OR=13.04; 95% CI, 2.77-61.46
COPD	OR = 3.70; 95% CI, 2.93-4.68	OR=9.2; 95% CI, 1.2-70.56	OR^138^ = 3.1; 95% CI, 2.6-4.2
Bilateral involvement of chest radiographs	OR=4.65; 95% CI, 2.97-7.34	OR=5.85 95% CI, 3.74-9.15	OR^138^ =4.89; 95% CI,1.16-20.47
**Liver function**			
CLD	OR = 1.48; 95% CI, 1.09-2.01	OR=25.35; 95% CI, 7.37-87.19	OR=2.36; 95% CI, 1.09-5.10
ALT, U/L	WMD = 7.11; 95% CI, 5.47-8.75	WMD = 10.08; 95% CI, 0.89-19.27	WMD = -3.07; 95% CI, -11.26-5.11
AST, U/L	WMD = 16.10; 95% CI, 14.00-18.20	WMD = 23.80; 95% CI, 11.32-36.29	WMD = 34.81; 95% CI, -4.39-74.02
Total bilirubin, mmol/L	WMD = 2.75; 95% CI, 2.11- 3.40	WMD = 6.31; 95% CI, 2.07 - 10.55	WMD = 7.09; 95% CI, 2.09-12.09

ALT, alanine aminotransferase; ARDS, acute respiratory distress syndrome; AST, aspartate aminotransferase; CI: confidence interval; CK: creatine kinase; CKD: chronic kidney diseases; CLD: chronic liver diseases; COPD, chronic obstructive pulmonary disease; CVD: cardiovascular diseases; LDH: lactate dehydrogenase; MERS: middle east respiratory syndrome ; OR: odds ratio; SARS: severe acute respiratory syndrome; WMD: weighted mean difference.

### Comparison of the impact of organ dysfunction on the severity of COVID-19, MERS, and SARS

We searched related articles to undertake a meta-analysis of SARS and MERS. The strategies were similar to COVID-19 [119-137].

CVD is a risk factor for predicting the severity of COVID-19, MERS, and SARS [138]. Similar to COVID-19 patients, LDH (WMD = 124.14 U/L; 95% CI, 31.09-217.19 U/L) and CK (WMD = 149.91 U/L; 95% CI, 94.03-205.79 U/L) levels of patients with severe SARS are frequently high. However, few studies have compared LDH and CK levels in patients with severe and non-severe MERS. As presented in Table 2, patients with either of the three viruses and renal and pulmonary dysfunction on admission were at high risk of developing severe disease. CLD was also a risk factor for the severity of SARS and MERS. No significant differences in ALT levels (WMD = -3.07 U/L; 95% CI, -11.26-5.11 U/L) or AST levels (WMD = 34.81 U/L; 95% CI, -4.39-74.02 U/L) was observed between patients with severe MERS and non-severe MERS (Table 2). Limited studies reported the relationship between neurologic dysfunction or GI dysfunction and SARS or MERS severity. Unlike SARS (*P*<0.001); [123] and COVID-19, chronic cerebrovascular diseases was not a predictor for the severity of MERS (OR = 1.94; 95% CI, 0.69-5.43); (Supplementary Fig. 2-6).

### Publications bias and Sensitivity analysis

Publication bias was noted in the following polled results: COPD (Begg=0.65, Egger=0.009), CKD (Begg=0.075, Egger=0.006), and headache (Begg=0.01, Egger=0.047) (Supplementary Table 2). The we performed trim-and-fill analysis. Although the direction of overall effects did not change, more articles should be included in future studies to reduce publication bias. Sensitivity analyses revealed no significant differences in the outcomes except for the pooled results of CLD, dizziness, nausea and/or vomiting and diarrhea (Supplementary Fig. 7).

## DISCUSSION

In the present study, data from 73 studies were meta-analyzed and the following conclusions were drawn. Although COVID-19 spreads rapidly and has a high incidence rate, it has a relatively low CFR and CSR. The most common comorbidity was hypertension, followed by diabetes and CVD. The most common complication was ARDS, followed by ACI and shock. Elevated levels of LDH and myoglobin were observed in COVID-19 patients. The most common neurologic symptom was olfactory and/or taste disorders and the most dominant GI symptom was anorexia. A history of organ dysfunction and abnormal biomedical indicators of organ function are significant predictors of a poor prognosis for all of the three coronavirus infections. In addition, patients with severe COVID-19 infections are more likely to have some neurologic and GI symptoms. Finally, acute organ injury was more commonly observed in severe COVID-19 cases.

According to studies on COVID-19, the presence of comorbidities is a predictor of a poor outcome. It is important to evaluate the prevalence of comorbidities in COVID-19 patients. In the present meta-analysis, the overall proportion of patients with hypertension, diabetes, CVD, chronic GI diseases, chronic cerebrovascular diseases, COPD, CLD, and CKD was 19%, 10%, 8%, 7%, 3%, 2%, 3%, and 3%, respectively. However, the prevalence of these chronic diseases is considerably higher in the general population [139, 140]. Hence, we could not determine whether patients with comorbidities are more susceptible to COVID-19 infection.

However, we assuredly do know that patients with comorbidities such as hypertension, CVD, CKD and COPD, are vulnerable to developing severe disease, which is consistent with previous studies [7, 141]. It can be speculated that comorbidities influence COVID-19 through two mechanisms. First, comorbidities are more prevalent among individuals of advanced age. Innate immunity serves as the first line of defense against pathogen invasion, and successful mounting of type I interferon (IFN) response should be able to suppress viral replication at an early stage [142]. Aging has been reported to impair the capacity of plasmacytoid dendritic cells, which are the most important type of I IFN producing cells [143, 144]. When the innate immune response is not effective and a delayed-type I IFN response is mounted, the virus cannot be controlled in the early phase of infection and patients are more likely to develop a severe infection. Second, in SARS and MERS infection, the influx of hyperinflammatory cytokines and chemokines is the main cause of lethal outcomes [144, 145]. Huang et al. reported that in COVID-19, higher levels of inflammatory factors were more commonly seen in patients with severe COVID-19 than in those with non-severe COVID-19, suggesting that a cytokine storm was associated with disease severity [8]. Patients with various chronic diseases are already in a proinflammatory state with impaired immune function; hence, the infection can accelerate the inflammatory progress [146]. Therefore, patients with a history of organ dysfunction may have a greater risk of developing severe COVID-19.

Another key finding is that COVID-19 appears to target multiple organs and induces acute injury. Li et al. found that the condition of patients without AKI at admission could gradually worsen and AKI may be diagnosed during hospitalization [147]. Guo et al. reported that some patients without underlying CVD had elevated TnT levels [148]. In this study, further analysis indicated that the incidence of acute multiorgan injury was considerably higher in patients with severe COVID-19. Similar to SARS-CoV, SARS-CoV-2 uses angiotensin-converting enzyme 2 (ACE2) as the cell entry receptor [149]. Zhao et al. demonstrated that ACE2 was principally expressed in alveolar epithelial type II cells, suggesting that the lung could be the most vulnerable target organ. However, ACE2 is highly expressed in the heart, kidney, liver, brain, and digestive tract, which provides a means for the involvement of multiple organs in COVID-19 [150-152]. Immunostaining of lung tissues indicated that the Rp3 nucleocapsid protein (NP) of SARS-CoV-2 was prominently expressed on alveolar epithelial cells, and histopathological examination revealed all the features of diffuse alveolar damage [103, 153]. Diao et al. found that various degrees of acute tubular necrosis and viral infection-associated syncytia were observed in renal specimens of COVID-19 patients. Furthermore, the NP antigens of SARS-CoV-2 could be observed in kidney tissues [154]. Clusters of coronavirus particles with distinctive spikes were observed in the tubular epithelium and podocytes [155]. No substantial histological changes were identified in the heart [103, 156], however SARS-CoV-2 viral particles have been identified in cardiac tissue in some COVID-19 cases [157, 158]. Biopsy of liver tissues revealed moderate microvesicular steatosis and mild lobular and portal activity; however, the possibility of drug-induced acute liver injury cannot be ruled out [103]. It has been reported that liver injury observed in COVID-19 patients after admission may be caused by LPV/r treatment [104]. Notably, viral RNA was also detected in the liver tissues of COVID-19 patients [158]. Similar to SARS-CoV, SARS-CoV-2 might invade the central nervous system via the nose close to the olfactory epithelium [159]. This was supported by the presence of olfactory disorders in patients with COVID-19. Detectable viral RNA was identified in the brain tissues of some COVID-19 cases [158], and the presence of specific SARS-CoV-2 RNA in COVID-19 patients’ cerebrospinal fluid has also been confirmed [160]. The SARS-CoV-2 RNA and NP were detected in GI tissues from COVID-19 patients [161]. In addition, the successful isolation of SARS-CoV-2 from stool samples demonstrated the fact that there was GI infection [162]. These results suggested that regional viral replication could directly contribute to acute organ damage. Of note, inflammatory cellular infiltration is commonly observed in multiple organs, including the lung, heart, kidney, and liver. This suggests that viruses not only induce direct organ damage but also aggravate the injury through proinflammatory function or cytokine storms [103, 156, 158]. Furthermore, the prevalence of a history of multiple organ dysfunction was also likely to partially explain the more frequent incidence of acute organ damage in severe COVID-19 patients. Besides, the effects of systemic hypoxia and abnormal coagulation could not be ruled out. According to our study results and the evidence from other studies, we strongly suggest that clinicians should be alert to the monitoring and protecting of extrapulmonary multiple organ function in COVID-19 patients in the early stage of infection.

In addition, clinicians should be equally alert to patients without organ dysfunction prior to admission. Guo et al. reported that patients with underlying CVD and normal TnT levels had a considerably more favorable outcome than in patients without CVD and with elevated TnT levels [148]. Therefore, it is reasonable to speculate that acute organ injury may play a greater role in the lethal outcome of COVID-19 than a history of organ dysfunction. These results indicate that clinicians should evaluate and prioritize COVID-19 patients according to the presence of organ dysfunction and the evidence of acute organ injury and initiate aggressive treatment. Multiorgan function biomarkers should be closely monitored for early warning and intervention.

SARS-CoV, MERS-CoV, and SARS-CoV-2 belong to the same genus and all are beta-CoVs. The comparison of genomic sequences revealed that the SARS-CoV-2 genome is 79% and 50% similar to SARS-CoV and MERS-CoV, respectively. SARS-CoV-2 contains additional gene regions, and the amino acid sequences of some SARS-CoV-2 proteins are only 68% similar to those of SARS-CoV [144, 163, 164]. It is reasonable to speculate that SARS-CoV-2 has similar clinical features and pathology as SARS-CoV and MERS-CoV; however, additional characteristics may yet be discovered. These findings reveal that all three coronaviruses might originate in bats prior to mutations. Subsequently they adapt to intermediate hosts and finally to humans [163, 165]. SARS-CoV and SARS-CoV-2 share the cell entry receptor of ACE2, while Dipeptidyl peptidase (DPP)-4 serves as the specific receptor for MERS-CoV [144, 166]. Similar to SARS and MERS, lower respiratory symptoms, including fever, cough, and fatigue, were commonly observed in patients with early-stage COVID-19, while the incidence of GI symptoms was relatively low [17, 141]. In the present study, a history of organ dysfunction was identified as the predictor of severe disease in all three coronaviruses. In addition, the pathological changes in the multiorgan tissues of COVID-19 patients significantly resemble those observed in SARS and MERS infection. This provides evidence for our work [167, 168]. Coronaviruses not only induce direct organ damage but also aggravate the injury through proinflammatory methods. On the basis of the information from previous studies, we found that SARS-CoV-2 shared similarities with SARS-CoV and/or MERS-CoV in various aspects, including genomic sequence, origin, entry receptor, clinical features, risk factors, and pathological processes. As no vaccine or specific treatment with confirmed results is available for treating COVID-19, and COVID-19 has a huge impact, clinical experience from SARS and MERS may provide some guidance regarding treatment strategies that may benefit COVID-19 patients.

### Limitations

Our meta-analysis has several limitations. First, most of the included studies are retrospective studies with a low level of evidence. However, the quality of the majority of studies included in the meta-analysis was moderate or high. Second, most patients in our meta-analysis were Chinese, and hence, conclusions might not be applicable to patients in different countries. Finally, some patients in the included studies remain in the hospital, which results in missing data.

### Conclusion

The present study successfully and systematically evaluated the relationship between multiorgan dysfunction and COVID-19 severity. Patients with a history of organ dysfunction have a greater risk of developing severe COVID-19 and in turn COVID-19 can promote acute multiorgan injury. Clinicians should increase their awareness regarding the monitoring of multiorgan function in hospitalized COVID-19 patients. Early detection and effective intervention to prevent multiorgan dysfunction may aid in reducing the number of deaths in these patients.

## Supplementary Materials

The Supplemenantry data can be found online at: www.aginganddisease.org/EN/10.14336/AD.2020.0520.


