# Supplementary material for: Multi-organ Dysfunction in Patients with COVID-19: A Systematic Review and Meta-analysis
Source: Aging Dis. 2020 May 13;11(4):874–94. doi: 10.14336/AD.2020.0520 (PMC7390520; doi:10.14336/AD.2020.0520)
Supplement: Supplementary file 1 [file AD-11-4-874-suppl.pdf]

SUPPLEMENTARY DATA

Multi-organ Dysfunction in Patients with COVID-19: A Systematic Review and Meta-analysis

Ting Wu[1,2], Zhihong Zuo[1,3], Shuntong Kang[1,3], Liping Jiang[3], Xuan Luo[4], Zanxian Xia[5,6], Jing Liu[1], Xiaojuan Xiao[1], Mao Ye[7], Meichun Deng[1,3,6,*]

[1]Department of Biochemistry and Molecular Biology & Hunan Province Key Laboratory of Basic and Applied Hematology, School of Life Sciences, Central South University, Hunan 410013, China. [2]Department of Cardiovascular Medicine, The Third Xiangya Hospital, Central South University, Changsha, Hunan 410013, China. [3]Xiangya School of Medicine, Central South University, Hunan 410013, China. [4]Hunan Yuanpin Cell Biotechnology Co., Ltd, Hunan 410129, China. [5]Department of Cell Biology, School of Life Sciences, Central South University, Changsha 410013, China. [6]Hunan Key Laboratory of Animal Models for Human Diseases, Hunan Key Laboratory of Medical Genetics & Center for Medical Genetics, School of Life Sciences, Central South University, Changsha 410013, China. [7]Molecular Science and Biomedicine Laboratory, State Key Laboratory for Chemo/Biosensing and Chemometrics, College of Biology, College of Chemistry and Chemical Engineering, Collaborative Innovation Center for Molecular Engineering for Theranostics, Hunan University, Changsha, China

© 2020. Wu T et al. Published online at http://www.aginganddisease.org/EN/10.14336/AD.2020.0520

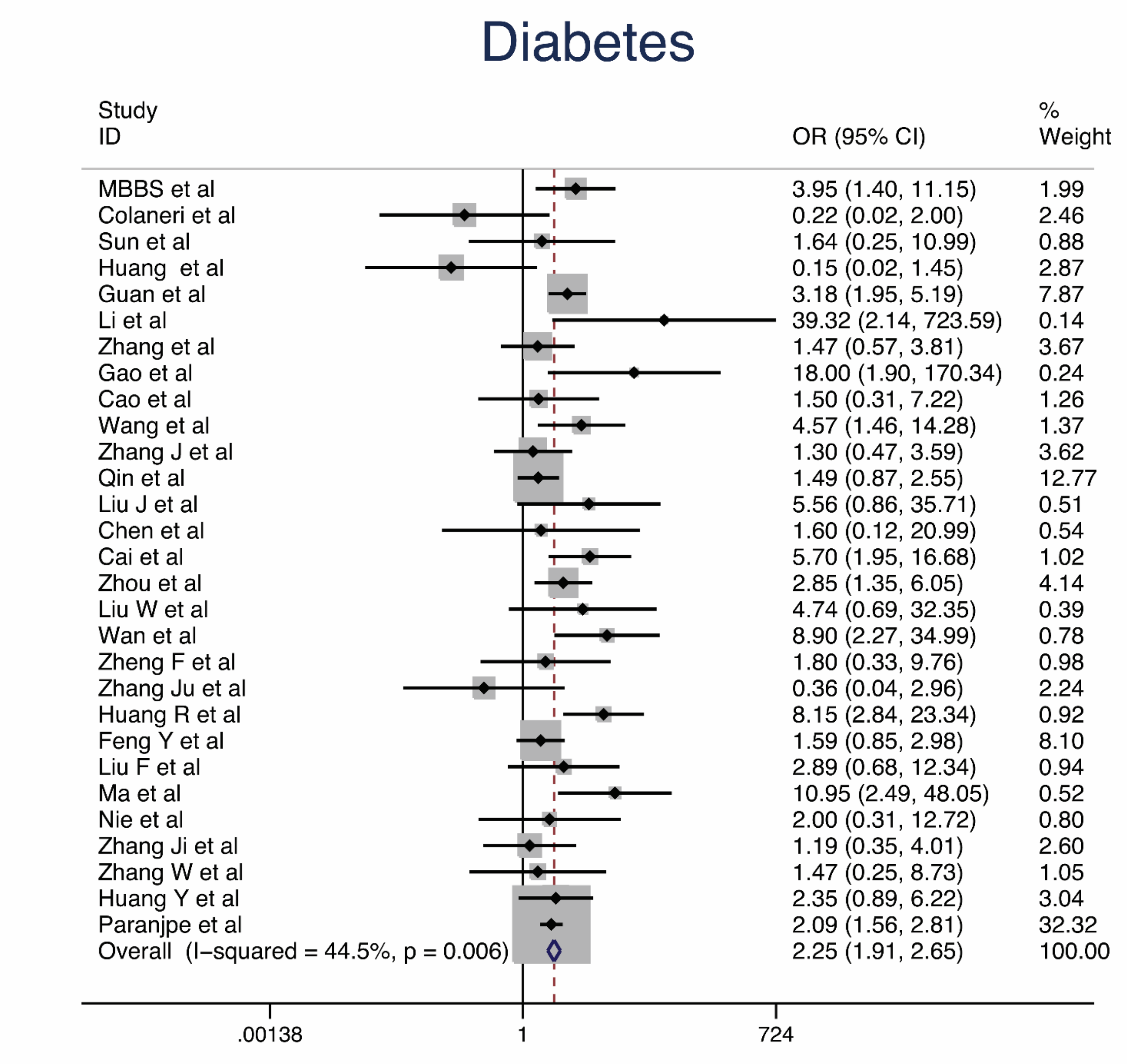

Supplementary Figure 1. Relationship between diabetes and COVID-19 severity.

© 2020. Wu T et al. Published online at http://www.aginganddisease.org/EN/10.14336/AD.2020.0520

SUPPLEMENTARY DATA

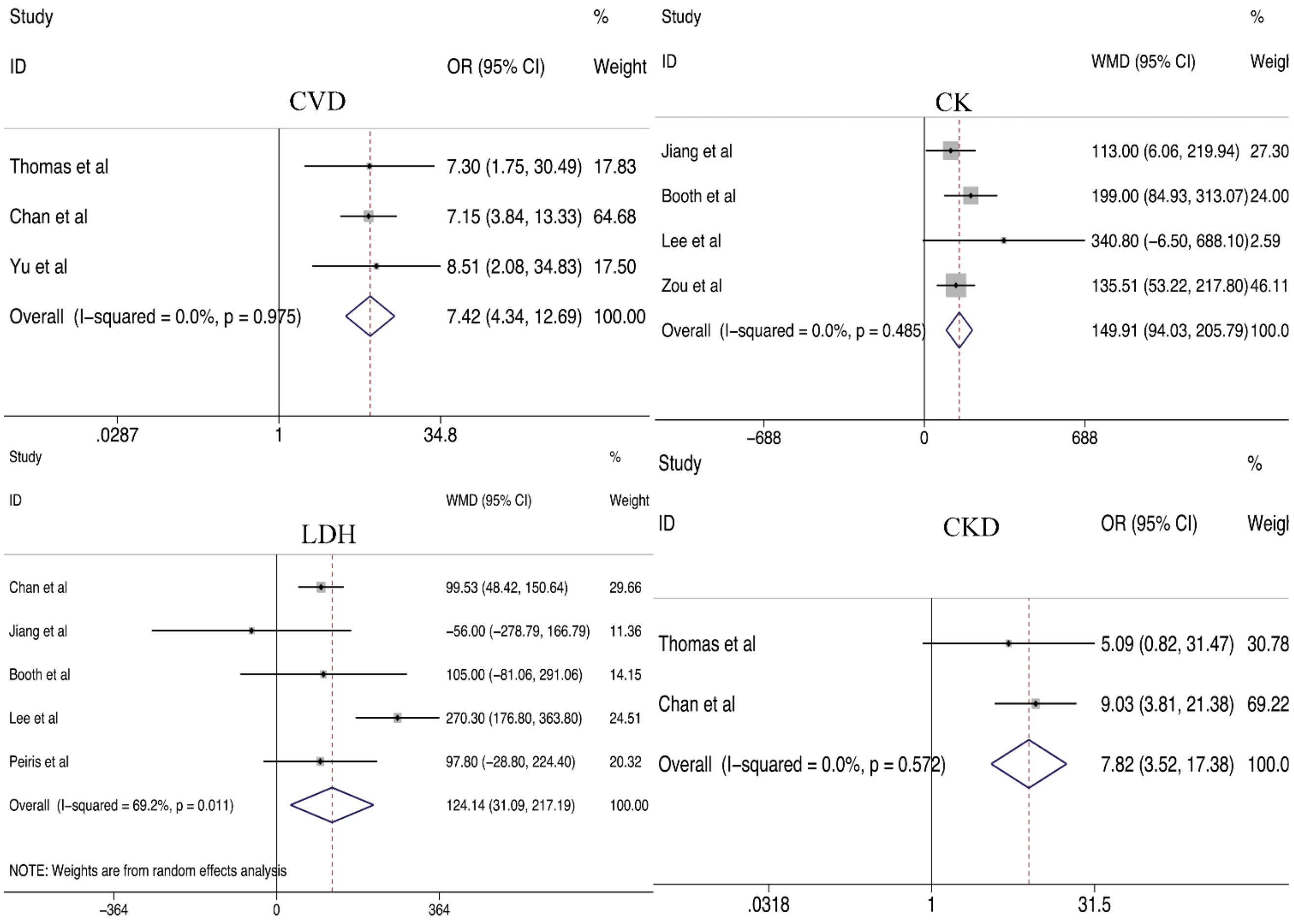

Supplementary Figure 2. Relationship between organ dysfunction (CVD, CK, LDH and CK) and SARS severity.

© 2020. Wu T et al. Published online at http://www.aginganddisease.org/EN/10.14336/AD.2020.0520

SUPPLEMENTARY DATA

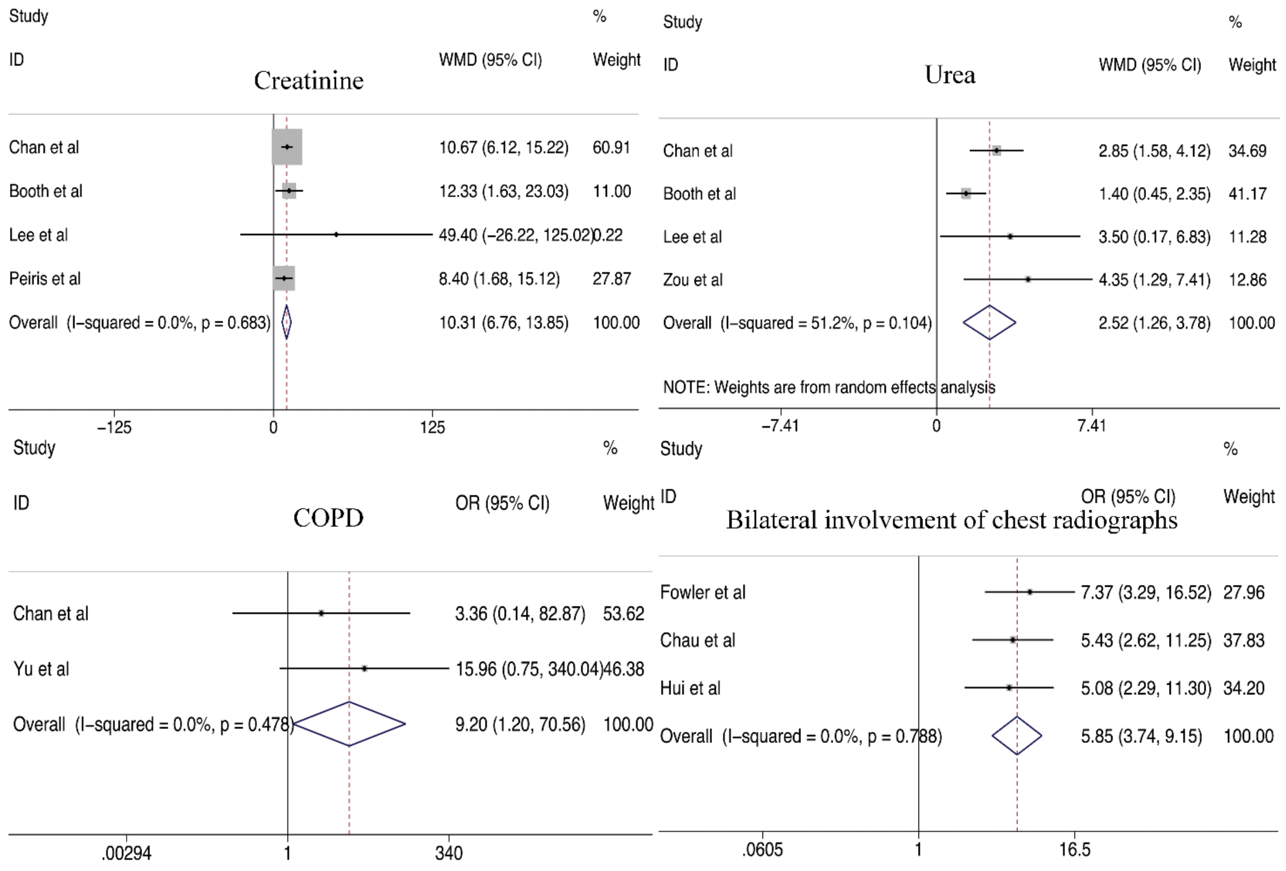

Supplementary Figure 3. Relationship between organ dysfunction (Creatinine, Urea, COPD and Bilateral involvement of chest radiographs) and SARS severity.

© 2020. Wu T et al. Published online at http://www.aginganddisease.org/EN/10.14336/AD.2020.0520

SUPPLEMENTARY DATA

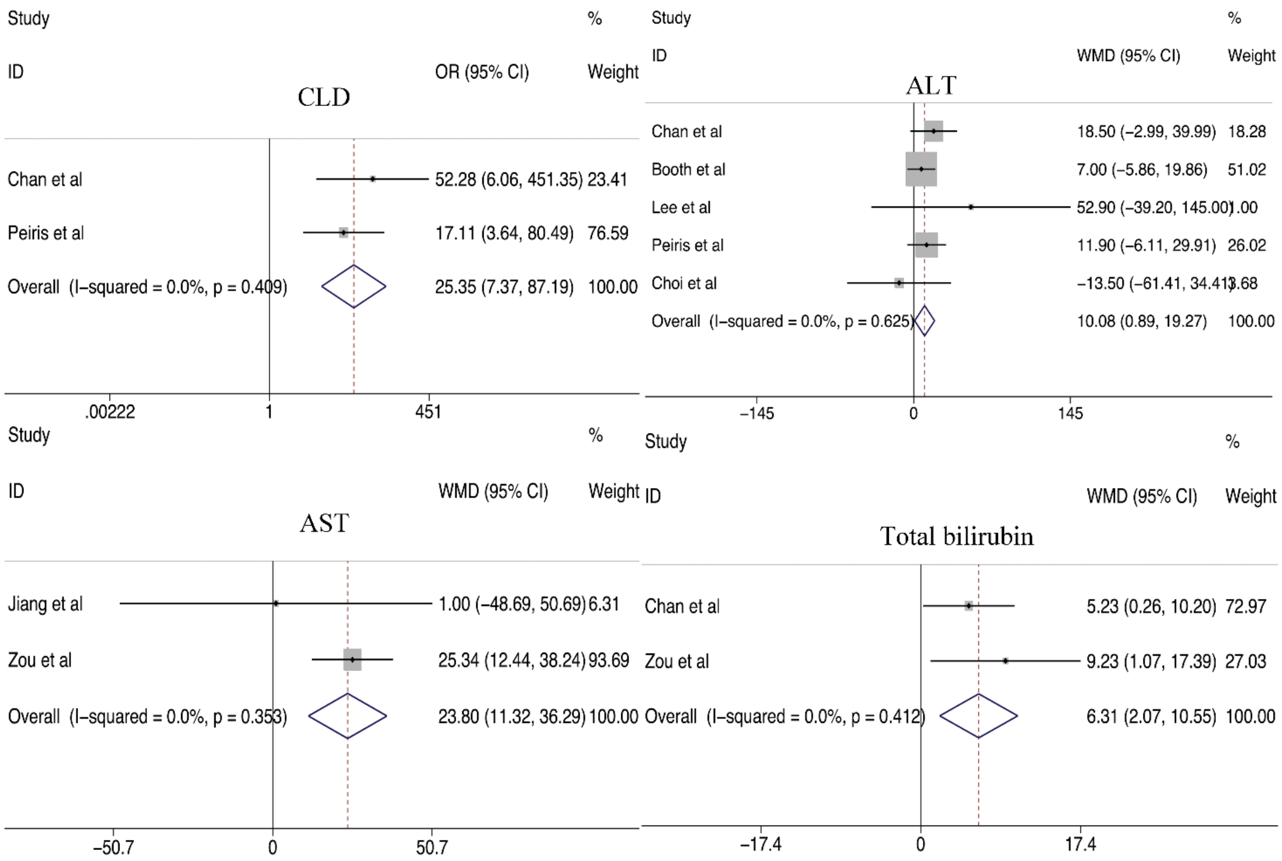

Supplementary Figure 4. Relationship between organ dysfunction (CLD, ALT, AST and Total bilirubin) and SARS severity.

© 2020. Wu T et al. Published online at http://www.aginganddisease.org/EN/10.14336/AD.2020.0520

SUPPLEMENTARY DATA

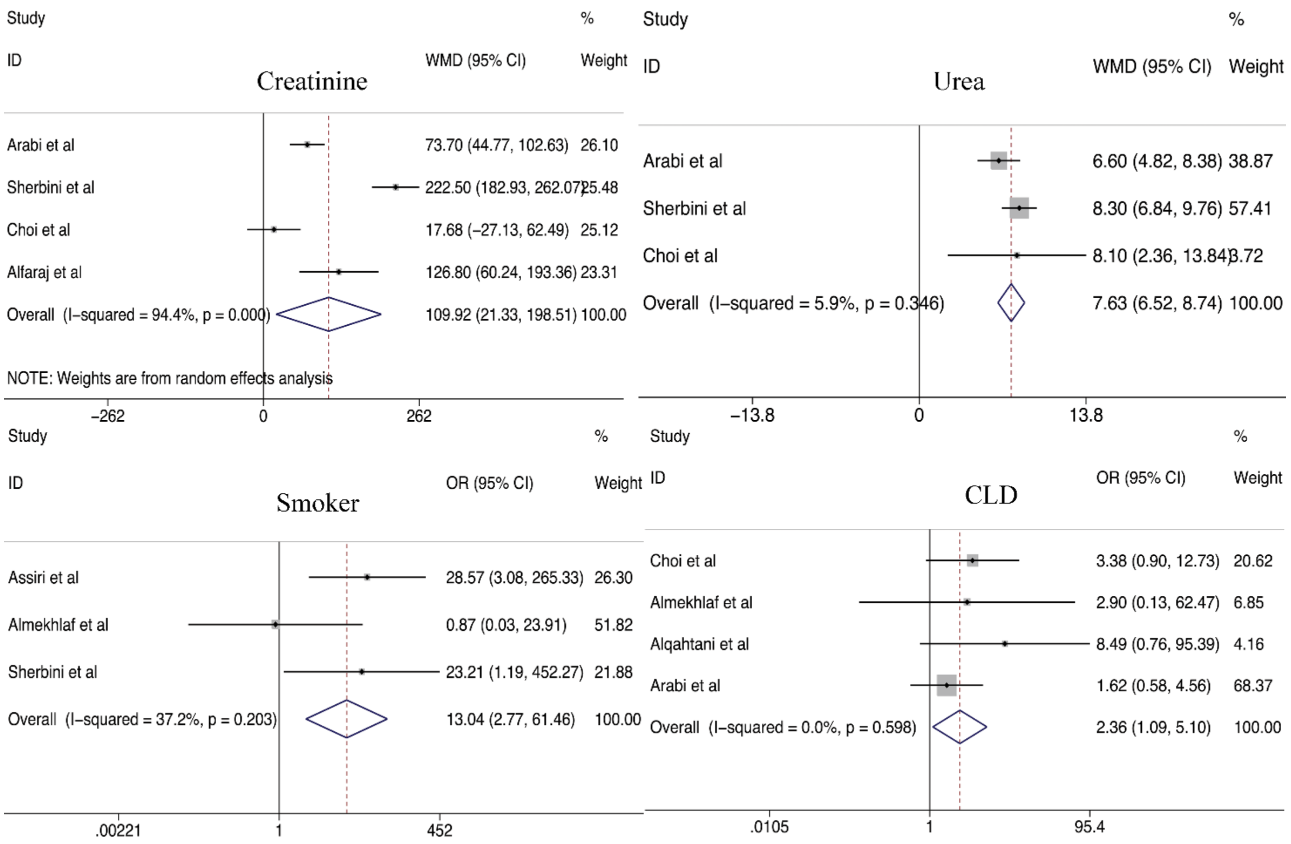

Supplementary Figure 5. Relationship between organ dysfunction (Creatinine, Urea, Smoker and CLD) and MERS severity.

© 2020. Wu T et al. Published online at http://www.aginganddisease.org/EN/10.14336/AD.2020.0520

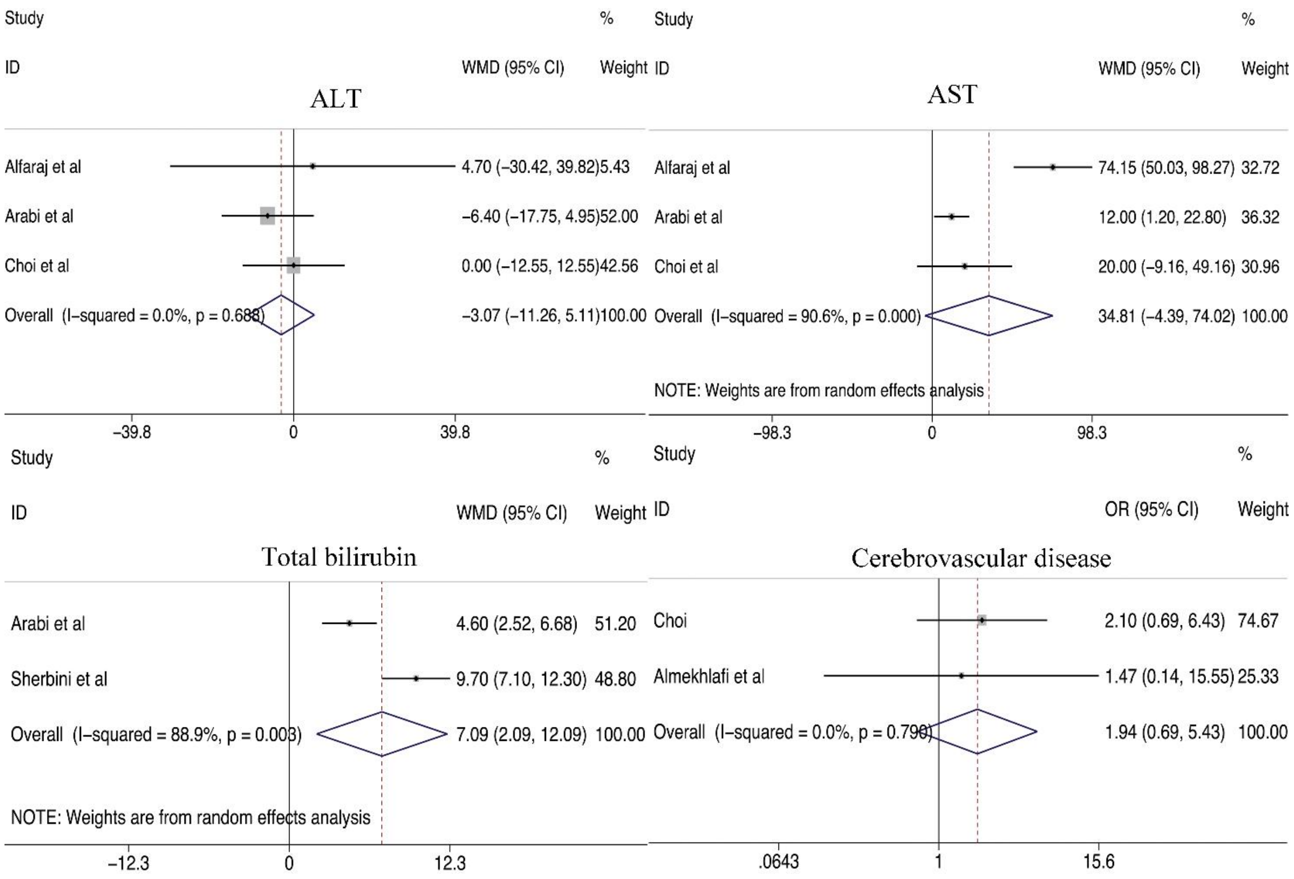

Supplementary Figure 6. Relationship between organ dysfunction (ALT, AST, Total bilirubin and Cerebrovascular disease) and MERS severity.

© 2020. Wu T et al. Published online at http://www.aginganddisease.org/EN/10.14336/AD.2020.0520

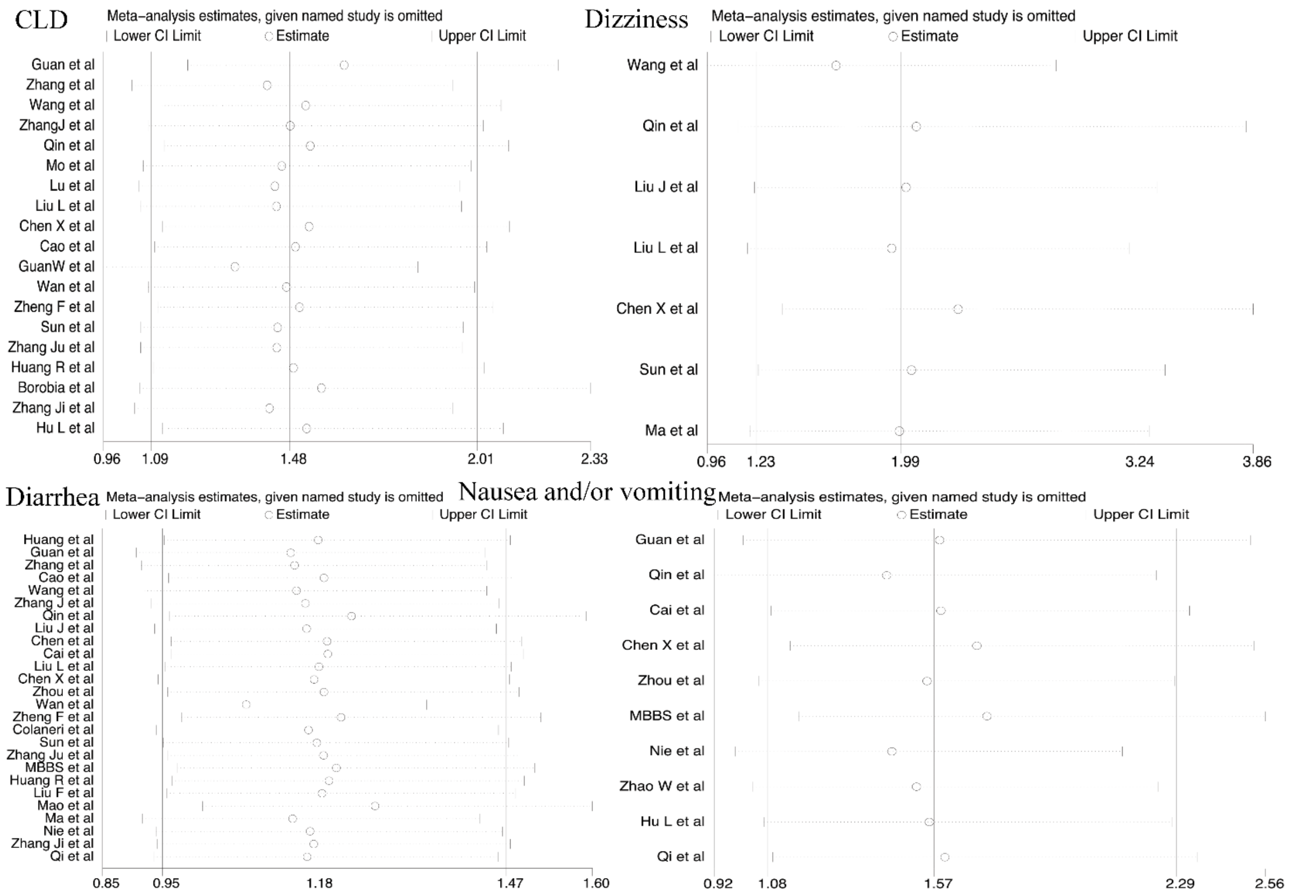

Supplementary Figure 7. Sensitivity analysis of outcomes: CLD, Dizziness, Nausea and/or vomiting and Diarrhea.

© 2020. Wu T et al. Published online at http://www.aginganddisease.org/EN/10.14336/AD.2020.0520

SUPPLEMENTARY DATA

Supplementary Table 1. Characteristics of included studies.

First author	Date	Country	Patients (N)	Average Age	Sex Male (%)	Research Type	Clinical symptoms	Quality
Huang et al [8]	Dec 16th 2019-Jan 2nd 2020	China	41	49.0	73.0	Retrospective study	Fever 98% / Cough 76%<br>Dyspnoea 55%<br>Myalgia/fatigue 44%	High
Zhao et al [15]		China	101	44.44	55.4	Retrospective study	Fever 78.2% / Cough 62.4%<br>Myalgia or fatigue 16.8%<br>Sore throat 11.9% / Dyspnea 1.0%	Moderate
Xu et al [16]	Jan-Feb 2020	China	50	43.9	58	Retrospective study	Fever 86% / Cough 40%<br>Fatigue 16% / Muscle ache 16%<br>Expectoration 14%	High
Guan et al [17]	Dec 11th 2019- Jan 29th 2020	China	1099	47	58.1	Retrospective study	Fever 88.7% / Cough 67.8%<br>Fatigue 38.1%<br>Sputum production 33.7%<br>Shortness of breath 18.7%	High
Liu et al [11]	Jan 21st- Feb 3rd 2020	China	73	41.6	56.2	Retrospective study	Fever 93 % / Cough 82%<br>Fatigue 75 % / Anorexia 27 %<br>Sputum production 53%	High
Li et al [18]	Jan-Feb 2020	China	83	45.5	53.0	Retrospective study	Fever 86.7% / Cough 78.3%<br>Expectoration 18.1%<br>Myalgia 18.1% / Headache 10.8%	High
Wu et al [40]	Jan 22th - Feb 14th 2020	China	80	46.1	48.75	Retrospective study	Fever 78.75% / Cough 63.75%<br>Shortness of breath 37.50%<br>Muscle ache 22.50%<br>Headache/mental disorder 16.25%	High
Qu et al[14]	Jan-Feb 2020	China	30	50.5	53.3	Retrospective study		Moderate
Zheng et al[30]		China	103	44.39	53.4	Retrospective study		Moderate
Qian et al[19]	Jan-Feb 2020	China	91	50	40.66	Retrospective study	Fever 71.43% / Cough 60.44%<br>Fatigue 43.96% /Anorexia 25.27%<br>Expectoration 32.97%	Moderate
Zhang et al [20]	Jan 2nd-Feb 10th 2020	China	221	55	48.9	Retrospective study	Fever 90.5% / Fatigue 70.6%<br>Cough 61.5% / Anorexia 36.2%<br>Dyspnea 29.0%	High
Qin et al [25]	Jan 10th - Feb 12th 2020	China	452	58	52.0	Retrospective study	Fever 92.6%<br>Shortness of breath 50.8%<br>Expectoration 41.4%<br>Fatigue 46.4%/Dry cough 33.3%	Moderate
Tabata etal [22]	Feb 11th- Feb 25th 2020	Japan	104	68	45.2	Retrospective study	Fever 34.6% / Cough 41.3%<br>Malaise 21.2%Tachypnea 23.1%<br>Runny nose 24.0%	Moderate
Cao et al [23]	Jan 20th - Feb 15th 2020	China	198	50.1	51.0	Retrospective study	Fever 86.9% / Cough 50.0%<br>Sputum production 27.8%<br>Myalgia or fatigue 29.8%	High

© 2020. Wu T et al. Published online at http://www.aginganddisease.org/EN/10.14336/AD.2020.0520

SUPPLEMENTARY DATA

							Headache or Dizziness 12.1%	
Wang et al [12]	Jan 1st-Jan 28th 2020	China	138	56	54.3	Retrospective study	Fever 98.6% / Fatigue 69.6%<br>Dry cough 59.4%/Myalgia 34.8%<br>Anorexia 39.9%	High
Zhang J et al[24]	Jan 16th -Feb 3rd 2020	China	140	57	50.7	Retrospective study	Fever 91.7% / Cough 75.0%<br>Fatigue 75.0%<br>Gastrointestinal symptoms 39.6%	High
Chen X et al [33]	Jan 23rd - Feb 14th 2020	China	291	46	49.8	Retrospective study	Fever 68.7% / Cough 60.5%<br>Fatigue 31.6% / Myalgia 9.3%<br>Headache 6.9%	High
Mo et al [26]	Jan 1st - Feb 5th 2020	China	155	54	55.5	Retrospective study	Fever 81.3%/ Cough 62.6%<br>Chest distress 39.4%<br>Fatigue 73.2%<br>Myalgia or arthralgia 61.0%	High
Liu J et al [27]	Jan 5th - Jan 24th 2020	China	40	48.7	37.5	Retrospective study	Fever 90.0% / Cough 82.5%<br>Sputum production 52.5%<br>Chill 25% / Fatigue 55%	Moderate
Chen et al [28]	Dec 19th 2019-Jan 27th 2020	China	21	56.3	81.0	Retrospective study	Fever 100% / Cough 80%<br>Fatigue 85% / Myalgia 40%<br>Sputum production 25%	High
Cai et al [29]	Jan 11th - Feb 6th 2020	China	298	47	50	Retrospective study	Fever 64.4% / Cough 18.1%<br>Fatigue 2.0% / Headache 1.3%<br>Dirrhea 2.0%	High
Lu et al [31]	Before Feb 7th 2020	China	265			Retrospective study	Fever 90.9% / Fatigue 25.3%<br>Cough 49.4%/ Inappetence 11.7%<br>Expectoration 23.0%	Moderate
Lie L et al [32]	Jan 20th - Feb 3rd 2020	China	51	45	62.7	Retrospective study	Fever 84.3% / Cough 74.5%<br>Fatigue 43.1% / Dyspnea 21.6%<br>Muscular soreness 11.8%	High
Gao et al [21]	Jan 23rd-Feb 2nd 2020	China	43	43.74	60.47	Retrospective study		Moderate
Liu W et al [41]	Dec 30th 2019- Jan 15th2020	China	78	38	50	Retrospective study	Cough 43.6%	Moderate
ZhangY et al [44]	Up to Feb 11th 2020	China	44672		51.4	Retrospective study		Moderate
Guan W et al[42]	Dec 30th 2019- Jan 31st2020	China	1590	48.9	57.3	Retrospective study	Fever 88%<br>Coughing 70.2%<br>Fatigue 42.8%<br>productive cough 36%	High
Yang et al [34]	Jan 17th - Feb 10th 2020	China	149	45.11	54.4	Retrospective study	Fever 76.5% / Cough 58.4%<br>Expectoration 32.2%<br>Sore throat 14.09% / Chill 14.09%	High
Chen N et al [35]	Jan 1st - Jan 20th 2020	China	99	55.5	67.7	Retrospective study	Fever 83% / Cough 82%<br>Shortness of breath 31%<br>Muscle ache 11% / Confusion 9%	High
Xu X et al [36]	Jan 10th - Jan 26th- 2020	China	62	41	56	Retrospective study	Fever 77%/ Myalgia/fatigue 52%<br>Cough 81% / Expectoration 56%	Moderate

© 2020. Wu T et al. Published online at http://www.aginganddisease.org/EN/10.14336/AD.2020.0520

SUPPLEMENTARY DATA

							Headache 34%	
Shi et al [37]	Dec 20th 2019-Jan 23rd 2020	China	81	49.5	52	Retrospective study	Fever 73% / Dyspnoea 42%<br>Cough 59% / Rhinorrhea 26%<br>Chest tightness 22%	Moderate
Zhou et al [38]	Dec 29th 2019- Jan 31st 2020	China	191	56	62	Retrospective study	Fever 94% / Cough 79%<br>Sputum 23% / Myalgia 15%<br>Fatigue 23%	High
Liu K et al [39]	Dec 30th 2019- Jan 24th 2020	China	137	57	44.5	Retrospective study	Fever 81.8% / Coughing 48.2%<br>Muscle pain or fatigue 32.1%<br>Dyspnea 19.0% / Headache 9.5%	Moderate
Wan et al [45]	Jan 23th-Feb 8th 2020	China	135	46	53.3	Retrospective study	Fever 88.9%<br>Cough 76.5%<br>myalgia or fatigue 32.5%<br>Headache 17.7%	High
Zhao W et al[48]	Jan 21st -Feb 8th 2020	China	77	52	44.2	Retrospective study	Fever 85.7% /Cough 63.7%<br>Fatigue 27.3%<br>Shortness of breath 20.8%<br>Headache or dizziness 13.0%	Moderate
YangYet al [43]	Up to Jan 26th 2020	China	4021	49	55	Retrospective study		Moderate
Ma et al[49]	Jan 21st-Mar2nd 2020	China	84	48	57.1	Retrospective study	Fever 64.3% /Cough 51.2%<br>Expectoration 33.3%<br>Fatigue 19.0%/Anorexia 16.7%	Moderate
Nie et al[50]	9th – 28th Feb 2020	China	97	39	35.1	Retrospective study	Fever 58.8% /Cough 55.7%<br>Fatigue 33%/ Diarrhea 12.45%<br>Sputum production 15.5%	High
Xu Xi et al [46]	Jan 23th-Feb 4th 2020	China	90	50	44.5	Retrospective study	Fever 78% / Coughing 63%<br>Fatigue 21%/Myalgia 28%<br>Sore throat 26%	Moderate
Qi et al[47]	Jan 19th - Feb 16th 2020	China	267	48	55.8	Retrospective study	Fever 84.3%/ Cough 70.8%<br>Fatigue 77.8%/ Dyspnea 16.2%<br>Nasal congestion 19.9%	High
Hu L et al[51]	Jan 8th - Feb 20th 2020	China	323	61	51.4	Retrospective study	Fever 83.9% Cough50.8%<br>Dyspnea 4.3% /Chest distress 0.9%<br>Headache 0.9%	High
Levinson et al[53]	Mar 10th-23rd 2020	Israel	42	34	44.2	Retrospective study	Cough 69% /Fatigue 69%<br>Myalgia or arthralgia 57%<br>Headache 48% /Dyspnea 43%	Moderate
Petrilli et al[52]	Mar 1st -Apr 2nd 2020	United States	4103			Retrospective study		High
Huang Y et al[54]	Jan 1st -Mar 8th 2020	China	223	62.0	56.5	Retrospective study	Fever 78.9% /Cough 69.1%<br>Fatigue 56.5%/Diarrhea 6.7%	Moderate
Fontanet et al[55]	Mar 30th- 4th Apr 2020	France	661	37	38.0	Retrospective study	Rhinitis 38.3% Cough 35.4%<br>Headache 30.9% /Asthenia 29.6%<br>Sore throat 26.8%	Moderate
Paranjpe et al[56]	Feb 27th - Apr 2nd 2020	United States	2199	65	58.8	Retrospective study		Moderate

© 2020. Wu T et al. Published online at http://www.aginganddisease.org/EN/10.14336/AD.2020.0520

SUPPLEMENTARY DATA

Buscarini et al[57]	Feb 21st- Mar 13th 2020	Italy	411		67.6	Retrospective study		Moderate
Docherty et al[58]	Feb 6th - Apr 18th 2020	United Kingdom	16749	72		Prospective study	Cough 70% /Fever 69% Shortness of breath 65%	Moderate
Borobia et al[59]	Feb 25th - Apr 19th 2020	Spain	2226	61	48.2	Retrospective study	Fever 71.2% /Cough 61.7% Dyspnea 49.8% /Myalgia 26.8% Diarrhea 21.7%	Moderate
Cabaraux Lechien et al[60]		France	86	41.7	34.9	Prospective study		High
Zhang Ji et al	Feb 1st and Mar 15th 2020	China	135	56	49.6	Retrospective study	Cough 100% /Fever 94.8% Fatigue 60.7% /Chest pain 34.1% Diarrhea 23.7%	Moderate
Ji Wo et al[61]	- Apr 8th 2020	South Korea	65149	44	48.3	Retrospective study		Moderate
Zheng F et al[63]	Jan 17th- Feb 7th 2020	China	161	45	49.7	Retrospective study	Fever 75.8% /Cough 62.7% Fatigue 39.8% /Dyspnea 14.3% Muscle ache 11.2%	Moderate
Wang Z et al[64]	Jan 16th - Jan 29th 2020	China	69	42	46	Retrospective study	Fever 87% /Cough 55% Fatigue 42%/Myalgia 30% Sputum production 29%	Moderate
Corbellini et al[67]	23rd – 25th Mar 2020	Spain	111	61.4	60.4	Prospective study		Moderate
Giacomelli et al[65]	Mar 19th 2020	Italy	59	60	67.8	Prospective study	Fever 72.8% /Cough 37.3% olfactory and/or taste disorders 33.9% Dyspnea 25.4%	Low
Luers et al[66]	22th - 28th Mar 2020	Germany	72	38	56.9	Retrospective study	Headache 77.8% / Sore Throat 62.5% Reduced olfaction 73.6% Muscle aches 70.8% /Cough 75%	Moderate
Lu Lu et al[68]	Jan 18th - Feb 18th 2020	China	304	44	59.9	Retrospective study		Moderate
Colaneri et al[69]	21th - 28th Feb 2020	Italy	44	/	63.6	Retrospective study		Moderate
Vaira et al[70]	Mar 31st - Apr 6th 2020	Italy	72	49.2	37.5	Prospective study	Fever 95.8% Cough 83.3% Asthenia 66.7%/Sore throat 51.4% Headache 41.6%	High
Mao et al[71]	Jan 16th -Feb 19th 2020	China	214	52.7	40.7	Retrospective study	Fever 61.7% Cough 60.0% Anorexia 31.8% /Diarrhea 19.2% Throat pain 14.5%	High
Sun et al[72]	Jan 20th -Feb 15th 2020	China	55	44	56.4	Retrospective study	Fever 81.8% Cough 47.3% Fatigue 25.5% /Expectoration 23.6% White sputum 16.4%	Moderate
Lechien et al[73]	Mar 22nd - Apr 10th 2020	France, Italy, Spain, Belgium, Switzerland	1566	39.17	32.3	Retrospective study	Headache 70.3%/Loss of smell 70.2% Nasal obstruction 67.8% Cough 63.2% Asthenia 63.3%	Moderate
Zhang Ya et al[74]	Jan 18th – Feb 22nd 2020	China	115	49.52	42.6	Retrospective study		Moderate
Zheng Y et al[75]	Jan 16th - Feb 20th 2020	China	99	49	52	Retrospective study		Moderate
Wang R et al[76]	Jan 20th - Feb 9th 2020	China	125	38.76	56.8	Retrospective study	Fever 92.8% /Cough 81.6% Shortness of breath 45.6% Sputum production 41.6%	High

© 2020. Wu T et al. Published online at http://www.aginganddisease.org/EN/10.14336/AD.2020.0520

Zhang Ju et al[77]	Jan 13th -Feb 16th 2020	China	111	38	41.4	Retrospective study	Fever 71.2% Cough 37.8% Fatigue 18.0% /Dyspnea 16.2% Chest pain 10.8%	High
Redd et al[78]	on or before Apr 2nd 2020	United States	318	63.4	54.7	Retrospective study	Fever 81.3% Fatigue 57.5% Myalgia 38.7% /Anorexia 34.8% Diarrhea 33.7%	Moderate
Tomlins MBBS et al[79]	10th -30th Mar 2020	United Kingdom	95	75	63	Retrospective study	Fever 72% /Cough 74% Shortness of breath 43% Confusion 21%/Myalgia 14%	Moderate
Huang R et al[80]	Jan 22nd – Feb 10th 2020	China	202	44	57.4	Retrospective study	Fever 77.2% Cough 59.4% Fatigue 21.8% /Sore throat 11.9% Muscle ache 10.4%	Moderate
Feng Y et al[81]	Jan 1st - Feb 15th 2020	China	476	53	56.9	Retrospective study	Fever 85.9% /Dry cough 59.4% Sputum production 35.5% Shortness of breath 24.4% Myalgia 12.6%	High
Menni et al[82]		United States United Kingdom	18401			Prospective study		Moderate
Liu F et al[83]	Jan 20th -Feb 3rd 2020.	China	134	51.5	47	Retrospective study	Fever 81.3% /Cough 39.6% Fatigue 19.4%/Poor appetite 9.7% Sputum production 17.9% Poor appetite 9.7%	High

Supplementary Table 2. Publication bias of indicators of multiorgan function and COVID-19 severity.

	Begg	Egger		Begg	Egger
Hypertension	0.195	1.479	CVD	0.116	0.117
Diabetes	0.896	0.551	CK	0.775	0.509
LDH	0.373	0.225	ALT	0.263	0.266
AST	0.902	0.911	Total bilirubin	0.300	0.899
Creatinine	0.661	0.719	Urea	0.474	0.430
ARDS	0.548	0.494	ACI	1.00	0.223
AKI	0.466	0.361	Smoker	0.152	0.268
COPD	0.65	0.009	CKD	0.075	0.006
Bilateral involvement of chest radiographs	0.101	0.120	CLD	0.234	0.561
Cerebrovascular disease	0.274	0.359	Headache	0.01	0.047
Anorexia	0.764	0.812	Dizziness	0.368	0.554
Abdominal pain	0.368	0.834	Diarrhea	0.537	0.629
Nausea and/or vomiting	0.371	0.909			

© 2020. Wu T et al. Published online at http://www.aginganddisease.org/EN/10.14336/AD.2020.0520
